# Computable upper error bounds for Krylov approximations to matrix exponentials and associated ${\varvec{\varphi }}$-functions

**DOI:** 10.1007/s10543-019-00771-6

**Published:** 2019-09-11

**Authors:** Tobias Jawecki, Winfried Auzinger, Othmar Koch

**Affiliations:** 1 Institut für Analysis und Scientific Computing, Technische Universität Wien, Wiedner Hauptstrasse 8–10/E101, 1040 Vienna, Austria; 2 Fakultät für Mathematik, Universität Wien, Oskar-Morgenstern-Platz 1, 1090 Vienna, Austria

**Keywords:** Matrix exponential, Krylov approximation, A posteriori error estimation, Upper bound, 15A16, 65F15, 65F60

## Abstract

An a posteriori estimate for the error of a standard Krylov approximation to the matrix exponential is derived. The estimate is based on the defect (residual) of the Krylov approximation and is proven to constitute a rigorous upper bound on the error, in contrast to existing asymptotical approximations. It can be computed economically in the underlying Krylov space. In view of time-stepping applications, assuming that the given matrix is scaled by a time step, it is shown that the bound is asymptotically correct (with an order related to the dimension of the Krylov space) for the time step tending to zero. This means that the deviation of the error estimate from the true error tends to zero faster than the error itself. Furthermore, this result is extended to Krylov approximations of $\varphi$-functions and to improved versions of such approximations. The accuracy of the derived bounds is demonstrated by examples and compared with different variants known from the literature, which are also investigated more closely. Alternative error bounds are tested on examples, in particular a version based on the concept of effective order. For the case where the matrix exponential is used in time integration algorithms, a step size selection strategy is proposed and illustrated by experiments.

## Introduction

We consider Krylov approximations to the matrix exponential function for the purpose of the solution of a linear, homogeneous system of differential equations

$$\begin{aligned} \psi '(t)=M\psi (t),\quad \psi (0)=\psi _0, \quad \psi (t) = \mathrm{e}^{tM} \psi _0. \end{aligned}$$

The complex-valued matrix *M* commonly results from the discretization of a partial differential equation. In this work we present new results for precise a posteriori error estimation, which also extend to the evaluation of so-called $\varphi$-functions. The application of these estimates for the purpose of time propagation is also discussed and illustrated. Theoretical results are verified by numerical experiments, which are classified into Hermitian (dissipative), skew-Hermitian (Schrödinger-type) and general non-normal problems.

*Overview of existing approaches and results* The approximate evaluation of large matrix exponential functions is a topic which has been extensively treated in the numerical analysis literature, for basic reference see e.g., [18, 35]. A standard approach is to project the given matrix *M* to a low-dimensional Krylov space via Arnoldi or Lanczos iteration, and to directly exponentiate the projected small matrix. A first mention of the Lanczos approach can be found in [42], where it is also recognized that for the method to perform satisfactorily, the time-steps have to be controlled. However, the control mechanism from [42] is not very elaborate and is based on a series expansion of the error, which is only valid in the asymptotic regime, see for instance [39]. For discretizations of parabolic problems, [17] uses an error estimator to choose the step-size, this approach is improved in [48] and has been generalized in [34]. Notably, in the latter reference a strict error bound is used to estimate the time-step instead of asymptotic techniques. It is argued in [34] that the strategy from [34] performs better than [33] and better in turn than [42].

A first systematic study of Krylov-based methods for the matrix exponential function was given in [45]. The error is analyzed theoretically, yielding both a priori and computable a posteriori estimates. The analysis there relies on approximation theory and yields a priori error bounds which are asymptotically optimal in the dimension of the Krylov subspace in important situations. The analysis moreover implies correction schemes to lift the convergence order which are cheap to compute based on the already available information. The error expansion also suggests a posteriori error estimators resorting to the leading error term. This approach relies on the assumption of the sufficiently rapid decay of the series representation of the error. A recent generalization of this work together with a more rigorous justification is given in [30]. For early studies of a priori error estimates see also [10, 12].

A thorough theoretical analysis of the error of Krylov methods for the exponential of a Hermitian or skew- (anti-) Hermitian matrix was given in [24]. The analysis derives an asymptotic error expansion and shows superlinear error decay in the dimension *m* of the approximation subspace for sufficiently large *m*. These results are further improved in [4]. In [24], a posteriori error estimation is also discussed. This topic is furthermore addressed in [31]. There, the Krylov approximation method is interpreted as a Galerkin method, whence an error bound can be obtained from an error representation for this variational approximation. This yields a computable estimate via a quadrature approximation of the error integral involving the defect of the numerical approximation. The a priori error analysis reveals a step-size restriction for the convergence of the method, which is less stringent when the subspace dimension is larger.

Further work in the direction of controlling the Lanczos process through information gained from the defect is given in [7]. The defect is a scalar multiple of the successive Krylov vector arising in the iteration and can be evaluated efficiently. If the error is approximated by a Galerkin approach, the resulting estimator corresponds to the difference of two Lanczos iterates. For the purpose of practical error estimation, in [7] it is seen as preferable to continue the original Krylov process. Some other defect-based upper bounds for the error of the matrix exponential are given in [30], including a closer analysis of the error estimate of [45]. These results still require some a priori information on the matrix spectrum.

Various improved methods for computing the matrix exponential function are given in the literature, for example restarted methods, deflated restarting methods or quadrature based restarting methods, see [1, 15, 16].

It has also been advocated in [49] to use preconditioning in the Lanczos method by a shifted inverse in order to get a good approximation of the leading invariant subspaces. The shift-and-invert approach (a specific choice to construct a rational Krylov subspace) for the matrix exponential function was introduced earlier in [37]. However, the choice of the shift is critical for the success of this procedure. This strategy amounts to a transformation of the spectrum which grants a convergence speed which is independent of the norm of the given matrix. In [49], a posteriori error estimation based on the asymptotical expansion of the error is advocated as well. We note that our results do not immediately carry over to the shift-and-invert approach, see Remark 3.

*Overview of present work* In Sect. 2 we introduce the Krylov approximation and the integral representation of the approximation error in terms of its defect. In Sect. 3 we derive a new computable upper bound for the error by using data available from the Krylov process with negligible additional computational effort (Theorem 1). This upper bound is cheap to evaluate and update on the fly during the Lanczos iteration. It is also asymptotically correct, i.e., for $t \rightarrow 0$ the error of the error estimator tends to zero faster asymptotically than the error itself. In Sect. 4 these results are extended to the case where the Krylov approach is employed to approximate the $\varphi$-functions of matrices (generalizing the exponential function), see Theorem 2. In Sect. 5, improved approximations derived from a corrected Krylov process [45] are discussed, and corresponding error estimators are analyzed, including an asymptotically correct true upper bound on the error (Theorem 3). This approach can be used to increase the order, but it has the drawback of violating mass conservation. In Proposition 6 error estimates are particularized to the Hermitian case. Another view on defect-based error estimation is presented in Sect. 6.

Section 7 is devoted to practical application of the various error estimators for the control of the time steps *t* including smaller substeps $\varDelta t$ if it appears indicated. In Sect. 8 we present numerical results for a finite difference discretization of the free Schrödinger equation, a Hubbard model of solar cells, the heat equation, and a convection–diffusion problem, illustrating our theoretical results. Additional practical aspects are also investigated: A priori estimates and the role of restarting are discussed in particular in the context of practical step-size adaptation. Finally, we demonstrate the computational efficiency of our adaptive strategy.

## Problem setting, Krylov approximation, and defect-based representation of the approximation error

We discuss the approximation of the matrix exponential,

2.1 $$\begin{aligned} E(t)v = \mathrm{e}^{\sigma \,t A} v, \quad A\in {\mathbb {C}}^{n\times n},~~\sigma \in {\mathbb {C}}, \end{aligned}$$

with step size *t*, applied to an initial vector $v \in {\mathbb {C}}^n$. To simplify the notation we assume $|\sigma |=1$ and $\Vert v\Vert _2=1$ without loss of generality. In many relevant applications (Schrödinger-type problems) a complex prefactor is applied to the matrix *A*. The parameter $\sigma$ is introduced here to separate the prefactor of the matrix *A*. The standard notation for Schrödinger-type problems is obtained in (2.1) with $\sigma =-\mathrm{i}$ and a Hermitian matrix *A*. For such problems our notation is helpful to simplify the construction of the Krylov subspace.

The exponential $E(t)=\mathrm{e}^{\sigma \,t A}$ satisfies the matrix differential equation

$$\begin{aligned} E'(t) = \sigma \,AE(t),\quad E(0)=I. \end{aligned}$$

We assume that $\mu _2(\sigma A)\le 0$, where $\mu _2(\sigma A)$ denotes the logarithmic norm of $\sigma A$, or equivalently, $W(\sigma A)\subseteq {\mathbb {C}}_-$, where $W(\sigma A)$ denotes the field of values of $\sigma A$ and we will refer to this assumption as the *nonexpansive case*. Following [23, Theorem 10.11] and associated references we conclude that $\mu _2(\sigma A)\le 0$ implies $\Vert E(t)\Vert _2\le 1$ for $t\ge 0$. This is essentially a technical assumption, and most of our theoretical results carry over to a more general setting, in particular if a priori information about $\mu _2(\sigma A)$ is available, such that *E*(*t*) can be estimated as $\Vert E(t)\Vert _2 \le \mathrm{e}^{\mu _2(\sigma A)}$.

For the skew-Hermitian case with $\sigma = -\mathrm{i}$ we write^1^

$$\begin{aligned} E(t)v = \mathrm{e}^{-\mathrm{i}\,t H} v, \quad H \in {\mathbb {C}}^{n\times n}~~\text {Hermitian.} \end{aligned}$$

In this case, *E*(*t*) represents a unitary evolution, i.e., $\Vert E(t)\Vert _2 = 1$.

*Krylov subspaces and associated identities* The numerical approximation of (2.1) considered here (see (2.6) below) is based on the conventional Krylov subspace

$$\begin{aligned} {\mathscr {K}}_m(A,v) = {{\,\mathrm{span}\,}}\{v,Av,\ldots ,A^{m-1}v\} \subseteq {\mathbb {C}}^n. \end{aligned}$$

First, an orthonormal basis of ${\mathscr {K}}_m(A,v)$ is obtained by the well-known Arnoldi iteration, see [46]. This produces a basis matrix $V_m \in {\mathbb {C}}^{n \times m}$ satisfying $V_m^*\,V_m = I_{m \times m}$, and an upper Hessenberg matrix $T_m \in {\mathbb {C}}^{m\times m}$ such that the Krylov identity^2^

2.2 $$\begin{aligned} A\,V_m = V_m T_m + \tau _{m+1,m}\,v_{m+1}e_m^*\end{aligned}$$

is valid, with $\tau _{m+1,m}\in {\mathbb {R}}_+$ and $v_{m+1}\in {\mathbb {C}}^n$ with $\Vert v_{m+1}\Vert _2=1$.

### Remark 1

We are assuming that the Arnoldi iteration is executed until the desired dimension *m*. Then, by construction, all lower diagonal entries of $T_m$ are positive [46]. If this is not the case, i.e., if a breakdown occurs, it is known that this breakdown is *lucky,*, i.e., the approximation (2.6) below obtained in the step before breakdown is already exact, see [45].

For the case of a Hermitian matrix *A* the Krylov subspace can be constructed using the Lanczos iteration, which is a special case of the Arnoldi iteration, resulting in a tridiagonal matrix $T_m \in {\mathbb {R}}^{m \times m}$. In the following we discuss the general case and comment on the case of a Hermitian matrix *A* whenever appropriate.

The following identities hold true due to the upper Hessenberg [tridiagonal] structure of $T_m$ together with (2.2):

2.3 $$\begin{aligned} e_m^*\,T_m^{j}\,e_1 = 0 \quad \text {for}\quad j=0,\ldots ,m-2, \end{aligned}$$

and

2.4 $$\begin{aligned} A^{j}v = V_m T_m^{j} e_1,\quad 0\le j \le m-1, \end{aligned}$$

see for instance [10, Theorem 2] or [45]. Furthermore, let

2.5 $$\begin{aligned} \gamma _m = e_m^*\,T_m^{m-1} e_1 = \prod _{j=1}^{m-1} (T_m)_{j+1,j}, \end{aligned}$$

where the claimed identity also follows from the upper Hessenberg [tridiagonal] structure of $T_m$.

*Krylov approximation* The standard Krylov approximation to *E*(*t*)*v* is

2.6 $$\begin{aligned} S_m(t)v = V_m\,\mathrm{e}^{\sigma \,t T_m}\,V_m^*\,v = V_m\,\mathrm{e}^{\sigma \,t T_m}e_1. \end{aligned}$$

We denote the corresponding error operator by $L_m(t)$, with

2.7 $$\begin{aligned} L_m(t)=E(t)-S_m(t) \in {\mathbb {C}}^{n \times n}. \end{aligned}$$

*Defect-based integral representation of the approximation error* We define the *defect*  (or residual) operator $D_m(t)$ of $S_m(t)$ by

$$\begin{aligned} D_m(t) = \sigma \,A\,S_m(t) - S_m'(t) \in {\mathbb {C}}^{n \times n}. \end{aligned}$$

Then, $L_m(t)v$ and $D_m(t)v$ are related via the differential equation

$$\begin{aligned} L_m'(t)v = \sigma \,A\,L_m(t)v + D_m(t)v, \quad L_m(0)v=0, \end{aligned}$$

whence

2.8 $$\begin{aligned} L_m(t)v = \int _0^t E(t-s)\,D_m(s)v\,\mathrm {d}s. \end{aligned}$$

An explicit representation for $D_m(s)v$ is obtained from (2.2),

2.9 $$\begin{aligned} D_m(s)v&= \sigma \,A\,V_m\,\mathrm{e}^{\sigma \,s\,T_m}e_1 - \sigma \,V_m T_m\,\mathrm{e}^{\sigma \,s\,T_m}e_1 = \sigma \,(A\,V_m - V_m T_m)\,\mathrm{e}^{\sigma \,s\,T_m}e_1\nonumber \\&= \sigma \tau _{m+1,m}\,\big (e_m^*\,\mathrm{e}^{\sigma \,s\,T_m}e_1\big )\,v_{m+1}. \end{aligned}$$

Asymptotically for $$ t \rightarrow 0 $$,

2.10 $$\begin{aligned} D_m(t)v = \sigma \tau _{m+1,m}\,\gamma _m\frac{(\sigma t)^{m-1}}{(m-1)!}\,v_{m+1} + {{\mathscr {O}}}(t^m), \end{aligned}$$

which follows from the Taylor series representation for $\mathrm{e}^{\sigma \,t\,T_m}$ together with (2.3) and (2.5). Thus, by (2.8) and (2.10) we obtain

2.11 $$\begin{aligned} \Vert D_m(t)v \Vert = {{\mathscr {O}}}(t^{m-1}), \quad \text {and} \quad \Vert L_m(t)v \Vert = {{\mathscr {O}}}(t^{m}). \end{aligned}$$

We can also characterize the asymptotically leading term of the error:

### Proposition 1

For any $A \in {\mathbb {C}}^{{n \times n}}$ the error $L_m(t)v$ satisfies the asymptotic relation

2.12 $$\begin{aligned} L_m(t)v = \tau _{m+1,m}\gamma _m\,\frac{(\sigma \,t)^{m}}{m!}v_{m+1} + R_{m+1}(t), \quad R_{m+1}(t) = {{\mathscr {O}}}(t^{m+1}), \end{aligned}$$

for $$ t \rightarrow 0 $$.

### Proof

Taylor expansion. Due to $L_m(t)v = {{\mathscr {O}}}(t^m)$, see (2.11),

2.13 $$\begin{aligned} \begin{aligned}&L_m(t)v = E(t)v - S_m(t)v = \frac{(\sigma \,t)^m}{{m!}}(A^m v - V_m T_m^m\,e_1) + R_{m+1}(t),\\&\text {with Taylor remainder}~~ R_{m+1}(t) = {{\mathscr {O}}}(t^{m+1}). \end{aligned} \end{aligned}$$

Multiplying the identity (2.4) (with $j = m-1$) by *A* and using (2.2) gives

$$\begin{aligned} A^m\,v&= A\,V_m T_m^{m-1} e_1 = (V_m T_m + \tau _{m+1,m}\,v_{m+1} e_m^*) T_m^{m-1} e_1 \\&= V_m T_m^m\,e_1 + \tau _{m+1,m}\, (e_m^*\,T_m^{m-1} e_1)\,v_{m+1} = V_m T_m^m\,e_1 + \tau _{m+1,m} \gamma _m v_{m+1}, \end{aligned}$$

whence (2.13) simplifies to (2.12). $\square$

### Remark 2

The Taylor remainder $R_{m+1}$ in (2.12) can be specified in a more explicit way showing its dependence on *m*,

$$\begin{aligned} R_{m+1}(t) = \frac{(\sigma \,t)^{m+1}}{m!} \int _{0}^{1} \big (A^{m+1} \mathrm{e}^{\sigma \,\theta \,t\,A} v - V_m T_m^{m+1}\mathrm{e}^{\sigma \,\theta \,t\,T_m} e_1 \big ){(1-\theta )}^m\,\mathrm {d}\theta . \end{aligned}$$

## An upper error bound for the nonexpansive case in (2.1)

For the nonexpansive case we have $\Vert E(t-s) \Vert _2 \le 1$ for $0\le s \le t$, and (2.8) implies

$$\begin{aligned} \Vert L_m(t)v\Vert _2 = \Big \Vert \int _0^t E(t-s)\,D_m(s)v\,\mathrm {d}s\ \Big \Vert _2 \le \int _0^t \Vert D_m(s)v\Vert _2\,\mathrm {d}s. \end{aligned}$$

With $$\Vert v_{m+1}\Vert _2 = 1$$, and

3.1a $$\begin{aligned} \delta _m(s) = e_m^*\,\mathrm{e}^{\sigma \,s\,T_m}e_1 = \big ( \mathrm{e}^{\sigma \,s\,T_m} \big )_{m,1}, \end{aligned}$$

together with (2.9) we obtain

3.1b $$\begin{aligned} \Vert L_m(t)v\Vert _2 \le \tau _{m+1,m} \int _0^t |\delta _m(s)| \, \mathrm {d}s. \end{aligned}$$

This estimate is also given in [31, Section III.2] and appeared earlier in [13, Subsection 2.2]. Of course, the integral in (3.1b) cannot be computed exactly. In [31] it is proposed to use numerical quadrature^3^ to approximate the integral in (3.1b). In contrast, our aim here is to derive a computable upper bound. We proceed in two steps.^4^

*Analytic matrix function via interpolation on the spectrum* To approximate the error integral in (3.1b) we use the representation of matrix exponentials via Hermite interpolation of the scalar exponential function on the spectrum of the matrix $T_m$, see [23, Chap. 1]: If $\mu _1,\ldots ,\mu _r$ ($r \le m$) denote the distinct eigenvalues of $T_m$ and $n_j$ is the dimension of the largest Jordan block associated with $\mu _j$, then

3.2 $$\begin{aligned} \mathrm{e}^{\sigma \,t T_m} = p_t(T_m), \end{aligned}$$

where $p_t(\lambda )$ is the Hermite interpolant of degree $\le m-1$ of the function

3.3 $$\begin{aligned} {f_t}(\lambda ) = \mathrm{e}^{\sigma \,t\lambda } \end{aligned}$$

over the nodes $\mu _1,\ldots ,\mu _m$ in the sense of [23, (1.7)],

$$\begin{aligned} p_t^{(\ell )}(\mu _j) = f_t^{(\ell )}(\mu _j), \quad j=1,\ldots ,r, \quad \ell =0,\ldots ,n_j-1. \end{aligned}$$

For a general matrix, the degree of $p_t$ may be smaller than $m-1$. However, in our context a special case occurs: Since the lower diagonal entries of $T_m$ do not vanish, $T_m$ is nonderogatory, i.e., for each eigenvalue $\mu _j$ the associated eigenspace is one-dimensional, see [27, Section 3.1]. Then, $\sum _{j=1}^{r} n_j=m$, which implies that the degree of $p_t$ is exactly $m-1$.

In the following we denote the full sequence of the *m* eigenvalues of $T_m$ by $\lambda _1,\ldots ,\lambda _m$. By applying basic properties of the Krylov decomposition and imposed conditions on the numerical range of $A$ we obtain

3.4 $$\begin{aligned} \text {spec}(\sigma \,T_m) \subseteq W(\sigma \,T_m) \subseteq W(\sigma \,A) \subseteq {\mathbb {C}}_-. \end{aligned}$$

The following proposition is partially related to [9, Sect. 3] or [30]. Here, divided differences have to be understood in the general sense, i.e., in the confluent sense if multiple eigenvalues occur; for the detailed definition and properties see [23, Section B.16].

### Proposition 2

Let $T_m \in {\mathbb {C}}^{m\times m}$ be an upper Hessenberg matrix with eigenvalues $\lambda _1,\ldots ,\lambda _m$ and $\text {spec}(\sigma T_m) \subseteq {\mathbb {C}}_-$. Then the function $\delta _m(t)$ defined as in (3.1a), i.e.,

$$\begin{aligned} \delta _m(t) = e_m^*\,\mathrm{e}^{\sigma \,t\,T_m}e_1 = \big ( \mathrm{e}^{\sigma \,t\,T_m} \big )_{m,1}, \end{aligned}$$

satisfies

3.5 $$\begin{aligned} \delta _m(t) = f_t[\lambda _1,\ldots ,\lambda _m] \gamma _m \le {\frac{t^{m-1}}{(m-1)!}\,\gamma _m,} \end{aligned}$$

with $\gamma _m$ from (2.5) and where $f_t[\lambda _1,\ldots ,\lambda _m]$ is the $(m-1)$-th divided difference over $\text {spec}(T_m)$ of the function $f_t$ defined in (3.3).

### Proof

We proceed from the Newton representation of the interpolant $p_t(\lambda )$ from (3.2),

$$\begin{aligned} p_t(\lambda ) = \sum _{j=0}^{m-1} f_t[\lambda _1,\ldots ,\lambda _{j+1}]\,\omega _j(\lambda ), \end{aligned}$$

with $\omega _j(\lambda ) = (\lambda -\lambda _1) \cdots (\lambda -\lambda _{j})$. From (2.3) and by definition of $\gamma _m$, it is obvious that the $\omega _j$ satisfy

$$\begin{aligned} e_m^*\,\omega _j(T_m)\,e_1 = \left\{ \begin{array}{ll} 0, &{}\quad j=0,\ldots ,m-2, \\ \gamma _m,&{}\quad j=m-1. \end{array}\right. \end{aligned}$$

Together with (3.2) this shows that the identity claimed in (3.5) is valid:

$$\begin{aligned} \delta _m(t)= & {} e_m^*\,\mathrm{e}^{\sigma \,t T_m}\,e_1 = e_m^*\,p_t(T_m) e_1 = \sum _{j=0}^{m-1} f_t[\lambda _1,\ldots ,\lambda _{j+1}] e_m^*\,\omega _j(T_m)e_1 \\= & {} f_t[\lambda _1,\ldots ,\lambda _m] \gamma _m. \end{aligned}$$

According to [23, (B.28)] the divided difference can be estimated by

$$\begin{aligned}&|f_t[\lambda _1,\ldots ,\lambda _m]| \le \frac{\max _{z \in \varOmega } D^{(m-1)}f_t(z)}{(m-1)!}\\&\text {for convex }\varOmega \subseteq {\mathbb {C}}\text { which contains all eigenvalues } \lambda _j. \end{aligned}$$

With $D^{(m-1)}f_t(\lambda ) = (\sigma \,t)^{m-1} \mathrm{e}^{\sigma \,t\lambda }$, $|\sigma |=1$ and $\text {Re}(\sigma \lambda _j)\le 0$ we obtain

$$\begin{aligned} |f_t[\lambda _1,\ldots ,\lambda _m]| \le \frac{t^{m-1}}{(m-1)!}, \end{aligned}$$

which implies the estimate (3.5) for $\delta _m(t)$. $\square$

*Error estimate and asymptotical correctness* Now we apply Proposition 2 in the context of our Krylov approximation.

### Theorem 1

(Computable upper bound) For the nonexpansive case the error $$L_m(t)v $$ of the Krylov approximation (2.6) to *E*(*t*)*v* satisfies

3.6 $$\begin{aligned} \Vert L_m(t)v \Vert _2 \le \tau _{m+1,m}\gamma _m\,\frac{t^{m}}{m!} \end{aligned}$$

with $\tau _{m+1,m}$ from (2.2) and $\gamma _m$ from (2.5).

### Proof

We proceed from (3.1). For $\delta _m$ defined in (3.1a), Proposition 2 implies

$$\begin{aligned} |\delta _m(s)| \le \frac{s^{m-1}}{(m-1)!} \gamma _m, \end{aligned}$$

and this gives an upper bound for the error integral (3.1b):

$$\begin{aligned} \Vert L_m(t)v\Vert _2 \le \tau _{m+1,m} \gamma _m \int _0^t \frac{s^{m-1}}{(m-1)!}\,\mathrm {d}s = \tau _{m+1,m}\gamma _m \frac{t^{m}}{m!}, \end{aligned}$$

which completes the proof. Proposition 2 is applied here in the nonexpansive case ($W(\sigma A) \subseteq {\mathbb {C}}_-$) which implies the requirement $\text {spec}(\sigma T_m) \subseteq {\mathbb {C}}_-$, see (3.4).$\square$

The upper bound (3.6) corresponds to the 2-norm of the leading error term (2.12) according to Proposition 1. It is easily computable from the Krylov decomposition (2.2). We denote the error estimate given by (3.6) as 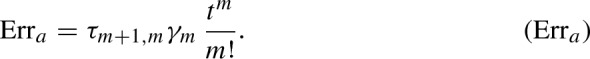


### Proposition 3

(Asymptotical correctness) The upper bound (3.6) is asymptotically correct for $t\rightarrow 0$, i.e.,

3.7 $$\begin{aligned} \Vert L_m(t)v \Vert _2 = \tau _{m+1,m}\gamma _m\,\frac{t^m}{m!} + {{\mathscr {O}}}(t^{m+1}). \end{aligned}$$

### Proof

The asymptotic estimate

$$\begin{aligned} \bigg | \Vert L_m(t)v \Vert _2 - \tau _{m+1,m}\gamma _m\,\frac{t^m}{m!} \bigg |&= \bigg | \Vert L_m(t)v \Vert _2 - \Big \Vert \tau _{m+1,m}\gamma _m\,\frac{(\sigma \,t)^{m}}{m!}v_{m+1} \Big \Vert _2 \bigg | \\&\le \Big \Vert L_m(t)v - \tau _{m+1,m}\gamma _m\,\frac{(\sigma \,t)^{m}}{m!}v_{m+1} \Big \Vert _2 = {{\mathscr {O}}}(t^{m+1}) \end{aligned}$$

is valid due to Proposition 1, and this proves (3.7). $\square$

### Remark 3

In [49, Section 4] a defect-based error formulation is given for the shift-and-invert Krylov approximation of the matrix exponential function. In contrast to the standard Krylov method, the defect is not of order $m-1$ for $t\rightarrow 0$ there. Hence, our new results do not directly apply to shift-and-invert Krylov approximations. A study of a posteriori error estimates for the shift-and-invert approach is a topic of future investigations.

## Krylov approximation to $\varphi$-functions

As another application we consider the so-called $\varphi$-functions, with power series representation

4.1a $$\begin{aligned} \varphi _p(z) = \sum _{k=0}^{\infty } \frac{z^k}{(k+p)!}, \quad p\ge 0. \end{aligned}$$

We have $\varphi _0(z) = \mathrm{e}^z$, and

4.1b $$\begin{aligned} \varphi _p(z) = \frac{1}{(p-1)!} \int _0^1 (1-\theta )^{p-1}\mathrm{e}^{\theta z}\,\mathrm {d}\theta , \quad p \ge 1. \end{aligned}$$

As the matrix exponential, $\varphi$-functions of matrices also appear in a wide range of applications, such as exponential integrators, see for instance [2, 25, 26, 39, 47]. Krylov approximation is a common technique to evaluate $\varphi$-functions of matrices applied to a starting vector,

4.2 $$\begin{aligned} \varphi _p(\sigma \,t A)v \approx V_m \varphi _p(\sigma \,t T_m) e_1,\quad p\ge 0. \end{aligned}$$

Since $\varphi$-functions are closely related to the matrix exponential, our ideas can be applied to these as well. We use the following notation for the error in the $\varphi$-functions:

4.3 $$\begin{aligned} L^p_m(t)v = \varphi _p(\sigma \,t\,A)v - V_m \varphi _p(\sigma \,t\,T_m) e_1. \end{aligned}$$

With (4.3) we generalize the previously used notation: $L_m(t) = L^0_m(t)$.

### Theorem 2

The error of the Krylov approximation (4.2) to $\varphi _p(\sigma \,t A)v$ with $p \ge 0$ satisfies

4.4a $$\begin{aligned} L^p_m(t)v = \tau _{m+1,m}\,\gamma _m \frac{(\sigma \, t)^m}{(m+p)!} \, v_{m+1} + {\mathscr {O}}(t^{m+1}). \end{aligned}$$

Furthermore, in the nonexpansive case its norm is bounded by

4.4b $$\begin{aligned} \Vert L^p_m(t)v \Vert _2 \le \tau _{m+1,m}\,\gamma _m\,\frac{t^{m}}{(m+p)!}, \end{aligned}$$

and this bound is asymptotically correct for $t \rightarrow 0$.

### Proof

For $p=0$ the result directly follows from Propositions 1, 3 and Theorem 1. We now assume $p \ge 1$. Via the series representation (4.1a) of $\varphi _p$ we can determine the leading term of the error in an analogous way as in Proposition 1:

$$\begin{aligned} \varphi _p(\sigma \,t A)v - V_m \varphi _p(\sigma \,t T_m) e_1&= \frac{(\sigma \,t)^m \,(A^m v - V_m T_m^m e_1)}{(m+p)!} + {\mathscr {O}}(t^{m+1}) \\&= \tau _{m+1,m}\,\gamma _m \frac{(\sigma \, t)^m}{(m+p)!} \, v_{m+1} + {\mathscr {O}}(t^{m+1}), \end{aligned}$$

which proves (4.4a).

Furthermore, proceeding from (4.1b) we obtain

$$\begin{aligned} \varphi _p(\sigma \,t A)v - V_m \varphi _p(\sigma \,t T_m) e_1&= \frac{1}{(p-1)!} \int _0^1 (1-\theta )^{p-1}\big ( \mathrm{e}^{\sigma \,\theta \,t A} v {-} V_m \mathrm{e}^{\sigma \,\theta \,t T_m} e_1 \big ) \,\mathrm {d}\theta \\&= \frac{1}{(p-1)!} \int _0^1 (1-\theta )^{p-1} L_m(\theta \,t)v \,\mathrm {d}\theta , \end{aligned}$$

with the error $L_m(t)v$ for the matrix exponential case. Now we apply Theorem 1 to obtain

$$\begin{aligned} \Vert \varphi _p(\sigma \,t A)v - V_m \varphi _p(\sigma \,t T_m) e_1 \Vert _2&\le \frac{1}{(p-1)!} \int _0^1 (1-\theta )^{p-1} \Vert L_m(\theta \,t)v\Vert _2 \,\mathrm {d}\theta \\&\le \tau _{m+1,m}\,\gamma _m\,\frac{t^m}{(p-1)!\,m!} \int _0^1 (1-\theta )^{p-1} \theta ^m \,\mathrm {d}\theta \\&= \tau _{m+1,m}\,\gamma _m\,\frac{t^{m}}{(m+p)!}, \end{aligned}$$

which proves (4.4b).$$\square $$

## Corrected Krylov approximation for the exponential and $\varphi$-functions

Let us recall the well-known error representation given in [45].

### Proposition 4

(See [45, Theorem 5.1]) With the $\varphi$-functions defined in (4.1), the error (2.7) can be represented in the form

5.1 $$\begin{aligned} L_m(t)v = \tau _{m+1,m}\,\sigma \,t \sum _{j=1}^{\infty } e_m^*\varphi _{j}(\sigma \,tT_m)\,e_1 (\sigma \,t A)^{j-1}v_{m+1}. \end{aligned}$$

In [45] it is stated that, typically, the first term of the sum given in Proposition 4, formula (${\mathrm {Err}}_{1}$), is already a good approximation to $L_m(t)v$. Analogously to [45, Section 5.2] we use the notation ${\mathrm {Err}}_{1}$ for the norm of this term, 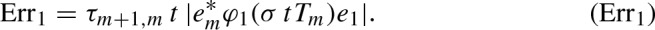


In [30] it is even shown that ${\mathrm {Err}}_{1}$ is an upper bound up to a factor depending on spectral properties of the matrix $A$. For the case of Hermitian $\sigma A$ we show $\Vert L_m(t)v\Vert _2 \le {\mathrm {Err}}_{1}$ in Proposition 6 below.

In Remark 4 below we show that ${\mathrm {Err}}_{1}$ is also an asymptotically correct approximation for the error norm (in the sense of Proposition 3). Furthermore, the error estimate ${\mathrm {Err}}_{1}$ is computable at nearly no extra cost, see [45, Proposition 2.1].

According to [45, Proposition 2.1], $\varphi _1(\sigma \,t T_m)$ can be computed from the extended matrix

5.2a $$\begin{aligned} {T}^+_m = \begin{bmatrix} T_m&\quad 0\\ \tau _{m+1,m}\,e_m^*&\quad 0 \end{bmatrix}\in {\mathbb {C}}^{(m+1)\times (m+1)} \end{aligned}$$

as

5.2b $$\begin{aligned} \mathrm{e}^{\sigma \,t{T}^+_m} e_1= \begin{bmatrix} \mathrm{e}^{\sigma \,tT_m}e_1\\ \tau _{m+1,m}\,\sigma \,t \,(e_m^*\,\varphi _1(\sigma \,t T_m)\,e_1) \end{bmatrix}\in {\mathbb {C}}^{m+1}. \end{aligned}$$

Equation (5.2b) can be used to evaluate the error estimate ${\mathrm {Err}}_{1}$ or a corrected Krylov approximation in the form

5.3 $$\begin{aligned} {S}^+_m(t)v = {V}^+_m\,\mathrm{e}^{\sigma \,t{T}^+_m}e_1\quad \text {with}\quad {V}^+_m=\Big [ V_m\,\big |\,v_{m+1} \Big ] \in {\mathbb {C}}^{n\times (m+1)}, \end{aligned}$$

for which the first term of the error expansion according to Proposition 4 vanishes, see [45]. For the error of the corrected Krylov approximation we use the notation

$$\begin{aligned} L_m^+(t)v = E(t)v - {S}^+_m(t)v. \end{aligned}$$

For general $\varphi$-functions we obtain an error representation similar to Proposition 4 and a corrected Krylov approximation for $\varphi$-functions. The corrected Krylov approximation to $\varphi _p(\sigma \,t\,A)v$ is given in [47, Theorem 2]:

$$\begin{aligned} \varphi _p(\sigma \,t\,A)v \approx {V}^+_m \varphi _p(\sigma \,t\,{T}^+_m) e_1 \end{aligned}$$

with ${T}^+_m$ and ${V}^+_m$ given in (5.2a) and (5.3). The error of the corrected Krylov approximation is denoted by

5.4 $$\begin{aligned} L^{p,+}_m(t)v = \varphi _p(\sigma \,t\,A)v - {V}^+_m \varphi _p(\sigma \,t\,{T}^+_m) e_1. \end{aligned}$$

### Proposition 5

(See [47, Theorem 2]) The error of the Krylov approximation $L^p_m(t)v$, see (4.3), satisfies

5.5a $$\begin{aligned} L^p_m(t)v = \tau _{m+1,m} \sigma \,t \sum _{j=p+1}^{\infty } (e_m^*\varphi _j(\sigma \,t\,T_m) e_1) \,(\sigma \,t\,A)^{j-p-1} v_{m+1}. \end{aligned}$$

The error of the corrected Krylov approximation $L^{p,+}_m(t)v$, see (5.4), is given by

5.5b $$\begin{aligned} L^{p,+}_m(t)v = \tau _{m+1,m} \sigma \,t \sum _{j=p+2}^{\infty } (e_m^*\varphi _j(\sigma \,t\,T_m) e_1) \,(\sigma \,t\,A)^{j-p-1} v_{m+1}. \end{aligned}$$

The following remark will be used later on.

### Remark 4

From the representation (4.1a) for the $\varphi _j$ together with (2.3) and (2.5) we observe

5.6 $$\begin{aligned} e_m^*\varphi _j(\sigma t T_m) e_1= & {} \frac{(\sigma t)^{m-1} e_m^*T_m^{m-1}\,e_1}{(m-1+j)!} + {\mathscr {O}}(t^m) = \gamma _m \,\frac{(\sigma t)^{m-1}}{(m-1+j)!} + {\mathscr {O}}(t^m).\nonumber \\ \end{aligned}$$

By (5.6) we observe $e_m^*\varphi _{j}(\sigma \,tT_m)\,e_1 = {\mathscr {O}}(t^{m-1})$ for $j \ge 0$ and we conclude that the asymptotically leading order term of $L^p_m(t)v$ for $t \rightarrow 0$ is obtained by the leading term ($j=p+1$) of the series (5.5a):

5.7a $$\begin{aligned} L^p_m(t)v = \tau _{m+1,m} \sigma \,t (e_m^*\varphi _{p+1}(\sigma \,t\,T_m) e_1 ) v_{m+1} + {\mathscr {O}}(t^m). \end{aligned}$$

Analogously we obtain the asymptotically leading order term of $L^{p,+}_m(t)v$ for $t \rightarrow 0$ by the leading term ($j=p+2$) of the series (5.5b):

5.7b $$\begin{aligned} L^{p,+}_m(t)v = \tau _{m+1,m} (\sigma \,t)^2 (e_m^*\varphi _{p+2}(\sigma \,t\,T_m) e_1) \,Av_{m+1} + {\mathscr {O}}(t^{m+1}). \end{aligned}$$

The asymptotically leading terms in (5.7a) and (5.7b) can be used as error estimators:

5.8a $$\begin{aligned} \Vert L^p_m(t)v\Vert _2 \approx \tau _{m+1,m}\,t |e_m^*\varphi _{p+1}(\sigma \,t\,T_m) e_1| \end{aligned}$$

and

5.8b $$\begin{aligned} \Vert L^{p,+}_m(t)v\Vert _2 \approx \Vert Av_{m+1}\Vert _2 \tau _{m+1,m}\,t^2 |e_m^*\varphi _{p+2}(\sigma \,t\,T_m) e_1|. \end{aligned}$$

The error estimators (5.8a) and (5.8b) are already suggested in [39, 47]. We will refer to them as ${\mathrm {Err}}_{1}$ in the context of the $\varphi$-functions with standard and corrected Krylov approximation, generalizing the corresponding quantities for the exponential case $p=0$.

We also obtain true upper bounds for the matrix exponential ($p=0$) and general $\varphi$-functions with $p \ge 1$.

### Theorem 3

The error of the corrected Krylov approximation (5.4) to $\varphi _p(\sigma \,t A)v$ with $p \ge 0$ satisfies

5.9a $$\begin{aligned} L^{p,+}_m(t)v = \tau _{m+1,m}\,\gamma _m \frac{(\sigma \, t)^{m+1}}{(m+p+1)!} \, Av_{m+1} + {\mathscr {O}}(t^{m+2}). \end{aligned}$$

Furthermore, in the nonexpansive case its norm is bounded by

5.9b $$\begin{aligned} \Vert L^{p,+}_m(t)v\Vert _2 \le \Vert Av_{m+1}\Vert _2\, \tau _{m+1,m}\,\gamma _m \frac{t^{m+1}}{(m+p+1)!}, \end{aligned}$$

and this bound is asymptotically correct for $t \rightarrow 0$.

### Proof

Applying (5.6) (with $j=p+2$) to (5.7b) shows (5.9a):

$$\begin{aligned} L^{p,+}_m(t)v = \tau _{m+1,m}\, \gamma _m\, \frac{(\sigma \,t)^{m+1}}{(m+p+1)!} \,Av_{m+1} + {\mathscr {O}}(t^{m+2}). \end{aligned}$$

From Proposition 5 we observe

$$\begin{aligned} L^{p,+}_m(t)v = \sigma \,t\,A\,\,L^{p+1}_m(t)v. \end{aligned}$$

Using the integral representation analogously as in the proof of Theorem 2 for $L^{p+1}_m(t)v$ and formula (2.8) for $L_m(t)v$, we obtain

$$\begin{aligned} L^{p,+}_m(t)v&= \sigma \,t\,A\,\, L^{p+1}_m(t)v = \tau _{m+1,m} \sigma \,t \frac{1}{p!} \int _0^1 (1-\theta )^{p} A\, L_m(\theta \,t)v \,\mathrm {d}\theta \\&= \tau _{m+1,m} \sigma \,t \frac{1}{p!} \int _0^1 (1-\theta )^{p} \int _0^{\theta t} \mathrm{e}^{\sigma (\theta t-s) A} Av_{m+1} \delta _m( s)\,\mathrm {d}s \,\mathrm {d}\theta . \end{aligned}$$

With norm inequalities (note the nonexpansive case) and Proposition 2 we obtain

$$\begin{aligned} \Vert L^{p,+}_m(t)v\Vert _2&\le \tau _{m+1,m} \,t\,\Vert Av_{m+1}\Vert _2 \frac{1}{p!} \int _0^1 (1-\theta )^{p} \int _0^{\theta t} |\delta _m( s)|\,\mathrm {d}s \,\mathrm {d}\theta \\&\le \Vert Av_{m+1}\Vert _2 \tau _{m+1,m} \gamma _m\, t^{m+1}\,\frac{1}{p!\,m!} \int _0^1 (1-\theta )^{p} \theta ^m\,\mathrm {d}s \,\mathrm {d}\theta \\&= \Vert Av_{m+1}\Vert _2 \tau _{m+1,m} \gamma _m \frac{t^{m+1}}{(m+p+1)!}, \end{aligned}$$

which proves (5.9b). Proposition 2 is applied here in the nonexpansive case, see also the proof of Theorem 1. $\square$

If the error estimate (5.9b) is to be evaluated, the effort of the computation of $\Vert Av_{m+1}\Vert _2$ is comparable to one additional step of the Krylov iteration.

As mentioned before, we also can show that for Hermitian $\sigma A$ the estimate ${\mathrm {Err}}_{1}$ gives a true upper bound:

### Proposition 6

For the nonexpansive case with $\sigma =1$ and a Hermitian matrix $A$ we obtain

$$\begin{aligned} |\delta _m(t)| = \delta _m(t)> 0\quad \text {for}\quad t > 0. \end{aligned}$$

This leads to the following upper bounds for the errors $L^p_m$ and $L^{p,+}_m$ with $p\ge 0$:

5.10a $$\begin{aligned} \Vert L^p_m(t)v\Vert _2 \le \tau _{m+1,m} \, t \, \underbrace{e_m^*\, \varphi _{p+1}(tT_m) \, e_1}_{\ge \, 0} \end{aligned}$$

and

5.10b $$\begin{aligned} \Vert L^{p,+}_m(t)v\Vert _2 \le \Vert Av_{m+1}\Vert _2 \,\tau _{m+1,m} \, t^2 \, \underbrace{e_m^*\, \varphi _{p+2}(tT_m) \, e_1}_{\ge \, 0}. \end{aligned}$$

### Proof

For a Hermitian matrix $A$ we obtain a symmetric, tridiagonal matrix $T_m$ with distinct, real eigenvalues via Lanczos approximation, see [27, Chap. 3.1]. By Proposition 2 we observe

$$\begin{aligned} \delta _m(t) = f_t[\lambda _1,\ldots ,\lambda _m] \gamma _m \end{aligned}$$

with $f_t(\lambda )=\mathrm{e}^{t\,\lambda }$ for the case $\sigma =1$. For divided differences of real-valued functions over real nodes we obtain $f_t[\lambda _1,\ldots ,\lambda _m] \in {\mathbb {R}}$ and

5.11 $$\begin{aligned} f_t[\lambda _1,\ldots ,\lambda _m] = \frac{D^{(m-1)} f_t(\xi )}{(m-1)!} = \frac{t^{m-1} \mathrm{e}^{t\xi }}{(m-1)!}\quad \text {for}\quad \xi \in [\lambda _1,\lambda _m]. \end{aligned}$$

Equation (5.11) shows $f_t[\lambda _1,\ldots ,\lambda _m] > 0$ and with $\gamma _m > 0$ we conclude

$$\begin{aligned} \delta _m(t) > 0,\quad \text {and}\quad |\delta _m(t)|=\delta _m(t). \end{aligned}$$

We continue with (5.10a) in the case $p=0$:

$$\begin{aligned}&\Vert L_m(t)v\Vert _2\le \tau _{m+1,m} \int _0^t |\delta _m(s)|\,\mathrm {d}s = \tau _{m+1,m} \int _0^t e_m^*\,\mathrm{e}^{sT_m} \,e_1 \,\mathrm {d}s \\&\quad = \tau _{m+1,m} t \, e_m^*\, \varphi _1(tT_m) \, e_1. \end{aligned}$$

For the case $p \ge 1$ we start analogously to Theorem 2. Using definition (4.1b) for the $\varphi$-functions and resorting to the case $p=0$ we find

$$\begin{aligned} \Vert L_m^p(t)v \Vert _2&\le \frac{1}{(p-1)!} \int _0^1 (1-\theta )^{p-1} \Vert L_m(\theta \,t)v\Vert _2 \,\mathrm {d}\theta \\&\le \tau _{m+1,m}\,t\,\frac{1}{(p-1)!} \int _0^1 (1-\theta )^{p-1} \theta \, e_m^*\, \varphi _1(\theta \,t\,T_m) \, e_1 \,\mathrm {d}\theta . \end{aligned}$$

Evaluation of the integral yields

5.12 $$\begin{aligned} \begin{aligned} \Vert L_m^p(t)v \Vert _2&\le \tau _{m+1,m}\,t\,\frac{1}{(p-1)!} \int _0^1 (1-\theta )^{p-1} \theta \, e_m^*\, \varphi _1(\theta \,t\,T_m) \, e_1 \,\mathrm {d}\theta \\&= \tau _{m+1,m}\,t\,\sum _{k=0}^{\infty }\frac{e_m^*\,(\,t\,T_m)^k \, e_1}{(p-1)!\,(k+1)!} \int _0^1 (1-\theta )^{p-1} \theta ^{k+1} \, \,\mathrm {d}\theta \\&= \tau _{m+1,m}\,t\,\sum _{k=0}^{\infty } \frac{e_m^*\,(\,t\,T_m)^k \, e_1}{(p+k+1)!}\\&= \tau _{m+1,m}\,t\,e_m^*\varphi _{p+1}(\,t\,T_m) \, e_1. \end{aligned} \end{aligned}$$

This shows (5.10a). To show (5.10b) we start analogously to Theorem 3:

$$\begin{aligned}&\Vert L^{p,+}_m(t)v\Vert _2 = \Vert t\,A\,L^{p+1}_m(t)v\Vert _2\\\le & {} \Vert Av_{m+1}\Vert _2\,\tau _{m+1,m} \,t\, \frac{1}{p!} \int _0^1 (1-\theta )^{p} \int _0^{\theta t} |\delta _m( s)|\,\mathrm {d}s \,\mathrm {d}\theta . \end{aligned}$$

Using $|\delta _m(s)|=\delta _m(s)$ and evaluating the inner integral by the $\varphi _1$ function, we obtain

$$\begin{aligned} \Vert L^{p,+}_m(t)v\Vert _2 \le \Vert Av_{m+1}\Vert _2\,\tau _{m+1,m}\,t^2\,\frac{1}{p!} \int _0^1 (1-\theta )^{p} \theta \, e_m^*\, \varphi _1(\theta \,t\,T_m) \, e_1 \,\mathrm {d}\theta . \end{aligned}$$

Evaluation of the integral analogously to (5.12),

$$\begin{aligned} \Vert L^{p,+}_m(t)v\Vert _2 \le \Vert Av_{m+1}\Vert _2\,\tau _{m+1,m}\,t^2\,e_m^*\varphi _{p+2}(\,t\,T_m) \, e_1, \end{aligned}$$

completes the proof. $\square$

## Defect-based quadrature error estimates revisited

The term on the right-hand side of (2.12) is a computable error estimate, which has been investigated more closely in Sect. 3. It can also be interpreted in an alternative way. To this end we again proceed from the integral representation (2.8),

6.1 $$\begin{aligned} L_m(t)v = \int _0^t \underbrace{E(t-s)\,D_m(s)}_{=:\;\varTheta _m(s,t)}v\,\mathrm {d}s. \end{aligned}$$

Due to $$ \Vert D_m(t)v \Vert = {{\mathscr {O}}}(t^{m-1}) $$,

$$\begin{aligned} \tfrac{\mathrm {d}^j}{\mathrm {d}\,s^j}\,D_m(s)v\big |_{s=0} = 0, \quad j=0,\ldots ,m-2, \end{aligned}$$

and the same is true for the integrand in (6.1),

$$\begin{aligned} \tfrac{\partial ^j}{\partial s^j}\,\varTheta _m(s,t)v\big |_{s=0} = 0, \quad j=0,\ldots ,m-2. \end{aligned}$$

Analogously as in [3], this allows us to approximate (6.1) by a Hermite quadrature formula in the form

6.2 $$\begin{aligned} \int _0^t \varTheta _m(s,t)v\,\mathrm {d}s \approx \frac{t}{m}\,\varTheta _m(t,t)v = \frac{t}{m}\,D_m(t)v. \end{aligned}$$

From (2.10),

$$\begin{aligned} \frac{t}{m}\,D_m(t)v = \tau _{m+1,m}\,\gamma _m\frac{(\sigma t)^{m}}{m!}\,v_{m+1} + {{\mathscr {O}}}(t^{m+1}), \end{aligned}$$

which is the same as (2.12). This means that the quadrature approximation (6.2) approximates the leading error term in an asymptotically correct way. From (6.2), (2.9) and (3.1a) we obtain

6.3 $$\begin{aligned} \Vert L_m(t)v\Vert _2 \approx \tau _{m+1,m}\frac{t}{m}|\delta _m(t)|. \end{aligned}$$

The quadrature error in (6.2) is ${{\mathscr {O}}}(t^{m+1})$. It is useful to argue this also in a direct way: By construction, the Hermite quadrature formula underlying (6.2) is of order *m*, and its error has the Peano representation (cf. also [3])

6.4 $$\begin{aligned} \frac{t}{m}\,\varTheta _m(t,t) - \int _0^t \varTheta _m(s,t)v\,\mathrm {d}s = \int _0^t \frac{s\,(t-s)^{m-1}}{m!}\, \tfrac{\partial ^{m}}{\partial s^{m}}\,\varTheta _m(s,t)v\,\mathrm {d}s. \end{aligned}$$

Here, $\tfrac{\partial ^{m}}{\partial s^{m}}\,\varTheta _m(s,t)v = {{\mathscr {O}}}(1)$, because $\tfrac{\mathrm {d}^{m}}{\mathrm {d}s^{m}}\,D_m(s)v = {{\mathscr {O}}}(1)$ which follows from $D_m(s)v = {{\mathscr {O}}}(s^{m-1})$. This shows that, indeed, the quadrature error (6.4) is ${{\mathscr {O}}}(t^{m+1})$. Furthermore, a quadrature formula of order $m+1$ can be constructed by including an additional evaluation of

$$\begin{aligned} \tfrac{\partial }{\partial s}\,\varTheta _m(s,t)v\big |_{s=t} = D_m^{[2]}(t)v, \quad \text {with} \quad D_m^{[2]}(t) = \tfrac{\mathrm {d}}{\mathrm {d}t} D_m(t) - \sigma A\,D_m(t). \end{aligned}$$

A routine calculation shows

6.5 $$\begin{aligned} \int _0^t \varTheta _m(s,t)v\,\mathrm {d}s = \frac{2\,t}{m+1}\,D_m(t)v - \frac{t^2}{m(m+1)}\,D_m^{[2]}(t)v + {{\mathscr {O}}}(t^{m+2}), \end{aligned}$$

where the error depends on $\tfrac{\mathrm {d}^{m+1}}{\mathrm {d}s^{m+1}}\,D_m(s)v = {{\mathscr {O}}}(1)$. This may be considered as an improved error estimate^5^ which can be evaluated using

$$\begin{aligned} \tfrac{\mathrm {d}}{\mathrm {d}t} D_m(t)v = \sigma ^2 \tau _{m+1,m}\,e_m^*(T_m\,\mathrm{e}^{\sigma \,t\,T_m})e_1 v_{m+1}. \end{aligned}$$

With the solution in the Krylov subspace, $\mathrm{e}^{\sigma \,t\,T_m} e_1$ with $e_m^*\mathrm{e}^{\sigma \,t\,T_m} e_1 = (\mathrm{e}^{\sigma \,t\,T_m} e_1)_m$, we can compute the derivative of the defect at ${\mathscr {O}}(1)$ cost,

$$\begin{aligned} \tfrac{\mathrm {d}}{\mathrm {d}t} D_m(t)v&= \sigma ^2 \tau _{m+1,m}\,e_m^*(T_m\,\mathrm{e}^{\sigma \,t\,T_m})e_1 v_{m+1} \\&= \sigma ^2 \tau _{m+1,m}\,\big ((T_m)_{m,m}\, (\mathrm{e}^{\sigma \,t\,T_m} e_1)_m + (T_m)_{m,m-1}\, (\mathrm{e}^{\sigma \,t\,T_m} e_1)_{m-1}\big )v_{m+1}. \end{aligned}$$

Also longer expansions may be considered, for instance

$$\begin{aligned} \int _0^t \varTheta _m(s,t)v\,\mathrm {d}s&= \frac{3\,t}{m+2}\,D_m(t)v - \frac{3\,t^2}{(m+1)(m+2)}\,D_m^{[2]}(t)v \\&\quad + \frac{t^3}{m(m+1)(m+2)}\,D_m^{[3]}(t)v + {{\mathscr {O}}}(t^{m+3}),\\&\quad \text {with} \quad D_m^{[3]}(t) = \tfrac{\mathrm {d}}{\mathrm {d}t} D_m^{[2]}(t) - A\,D_m^{[2]}(t), \end{aligned}$$

etc. This alternative way of computing improved error estimates is worth investigating but will not be pursued further here.

*Quadrature estimate for* (3.1b) *revisited* We proceed from (3.1b) which is valid for the nonexpansive case. In [31] it is suggested to use the right-endpoint rectangle rule as a practical approximation to the integral (3.1b),

6.6 $$\begin{aligned} \Vert L_m(t)v\Vert _2 \le \tau _{m+1,m} \int _0^t |\delta _m(s)| \, \mathrm {d}s \approx \tau _{m+1,m}\, {t} |\delta _m(t)|, \end{aligned}$$

or alternatively the Simpson rule, which is also suggested in [50]. The error estimate in (6.6) is also referred to as *generalized residual estimate*  in [7, 25] and similar error estimates also appeared earlier in [45]. In [13, eq. (32)] an a priori upper bound on the integral in (6.6) is obtained by $t \max _{s\in [0,t]}|\delta _m(s)|$. Applying Hermite quadrature to (3.1b) also directly leads to the error estimate (6.3).

For a better understanding of the approximation (6.6) we consider the effective order of $|\delta _m(t)|$ as a function of *t*. Let us denote $f(t):=|\delta _m(t)|$ and assume $f(t)>0$ in a sufficiently small interval (0, *T*]. For the Hermitian case this assumption is fulfilled for all $t>0$, see Proposition 6.

The effective order of the function *f*(*t*) can be understood as the slope of the double-logarithmic function

$$\begin{aligned} \xi (\tau )&=\ln (f(\mathrm{e}^{\tau }))\quad \text {with}\quad \tau =\ln t, \\ \text {with derivative} \quad \xi '(\tau )&=\frac{f'(\mathrm{e}^{\tau })\,\mathrm{e}^{\tau }}{f(\mathrm{e}^{\tau })}. \end{aligned}$$

We denote the order by

6.7 $$\begin{aligned} \rho (t)=\frac{f'(t)\,t}{f(t)}, \end{aligned}$$

and obtain

$$\begin{aligned} f(t)=\frac{f'(t)\,t}{\rho (t)}. \end{aligned}$$

Integration and application of the mean value theorem shows the existence of $t^*\in [0,t]$ with

$$\begin{aligned} \int _0^t f(s)\,\mathrm {d}s= \frac{1}{\rho (t^*)} \int _0^t f'(s)\,s\,\mathrm {d}s, \end{aligned}$$

and integration by parts gives

$$\begin{aligned} \int _0^t |\delta _m(s)| \,\mathrm {d}s = \frac{t\, |\delta _m(t)|}{1+\rho (t^*)}. \end{aligned}$$

With the plausible assumption that the order is bounded by ${0}\le {\tilde{m}}\le \rho (t) \le m-1 =\rho (0+)$ for $t\in [0,T]$, we obtain

6.8 $$\begin{aligned} \tfrac{t}{m}\,|\delta _m(t)| \le \int _0^t |\delta _m(s)| \,\mathrm {d}s \le \tfrac{t}{{\tilde{m}}+1}\,|\delta _m(t)| \le {t}\,|\delta _m(t)|. \end{aligned}$$

This shows that under such an assumption the generalized residual estimate (6.6) gives an upper bound on the error. With the assumption $0\le \rho (t)$, the defect $|\delta _m(t)|$ (also called the residual in the literature) is monotonically increasing and the upper bound suggested in [13, eq. (32)] is equivalent to the generalized residual estimate. However, in contrast to (6.3), the error estimate (6.6) is not asymptotically correct for $t \rightarrow 0$. In the following remark we suggest a practical approach to tighten the generalized residual estimate retaining the property of an upper bound in (6.8).

### Remark 5

With $\rho (0+)=m-1$ and the assumptions that the effective order is slowly decreasing locally at $t=0$ and sufficiently smooth, we suggest choosing ${\tilde{m}}=\rho (t)$ for a step of size *t* to improve the quadrature based estimate.

6.9 $$\begin{aligned} \Vert L_m(t)v\Vert _2 \le \tau _{m+1,m} \int _0^t |\delta _m(s)| \, \mathrm {d}s \approx \tau _{m+1,m} \tfrac{t}{\rho (t)+1} \,|\delta _m(t)|. \end{aligned}$$

We will refer to this as *effective order quadrature*  estimate. Substitute $f(t)=|\delta _m(t)|= \big ((\mathrm{e}^{\sigma \,t\,T_m}e_1)_m(\mathrm{e}^{{\bar{\sigma }}\,t\,{\bar{T}}_m}e_1)_m\big )^{1/2}$ in the ansatz (6.7) to obtain a computable formula for the effective order $\rho (t)$,

6.10 $$\begin{aligned} \rho (t)= \frac{t\,\big (|\delta _m(t)|\big )'}{|\delta _m(t)|} =t\, \text {Re} \bigg ( \sigma (T_m)_{m,m} + \sigma (T_m)_{m,m-1} \frac{(\mathrm{e}^{\sigma \,t\,T_m}e_1)_{m-1}}{(\mathrm{e}^{\sigma \,t\,T_m}e_1)_m}\bigg ). \end{aligned}$$

The computation of $(\mathrm{e}^{\sigma \,t\,T_m}e_1)_{m-1}$ usually comes hand in hand with the computation of $\delta _m(t)=(\mathrm{e}^{\sigma \,t\,T_m}e_1)_m$. For a numerical implementation of (6.10) we suggest computing $\mathrm{e}^{\sigma \,t\,T_m}e_1$ by a Taylor or Páde approximation. In the limit $t\rightarrow 0$ this choice of quadrature is equivalent to the Hermite quadrature and, therefore, asymptotically correct.

Up to now we did refer to the effective order of the defect $|\delta _m(t)|$. For $t \rightarrow 0$ the effective order of the error is given by $\rho (t)+1$.

## The matrix exponential as a time integrator

For simplicity we assume the nonexpansive case of (2.1) in this section.

We recall from [24] that superlinear convergence as a function of *m*, the dimension of the underlying Krylov space, sets in for

7.1 $$\begin{aligned} t\, \Vert A\Vert _2 \lessapprox m. \end{aligned}$$

This relation also affects the error considered as a function of time *t*. Equation (7.1) can be seen as a very rough estimate for a choice of *t* which leads to a systematic error and convergence behavior. Only for special classes of problems as for instance symmetric negative definite matrices, the relation (7.1) can be weakened, see [4, 24] for details.

In general a large time step *t* would necessitate large *m* or a restart of the Krylov method. For larger dimensional problems memory issues can limit the choice of *m* and make a restart necessary. Considering global computational cost it may also be favorable to use a moderate value of *m* in combination with restarts. Even if increasing *m* results in a larger time step *t*, the increase in computational cost can lead to a decrease of total performance in some cases. This issue is relevant particularly for rather large choices of *m*, especially if computational cost scaling with $m^2$ or worse gets noticeable. We further discuss effects of computer arithmetic on the Krylov approximation of the matrix exponential in Sect. 8 without going into details.

For the matrix exponential seen as a time propagator, a simple restart is possible. The following procedure has been introduced in [47] and is recapitulated here to fix the notation.

We split the time range [0, *t*] into *N* subintervals,

$$\begin{aligned} 0&=t_0<t_1<\cdots <t_N=t, \\ \text {with step sizes}~~\varDelta t_j&= t_{j}-t_{j-1},\quad j=1,\ldots ,N. \end{aligned}$$

The exact solution at time $t_j$ is denoted by $v^{[j]}$, whence

$$\begin{aligned} v^{[j]} = E(\varDelta t_{j})v^{[j-1]}=E(t_{j})v,\quad \text {with}\quad v^{[0]}=v. \end{aligned}$$

For simplicity we assume that the dimension *m* of the Krylov subspace is fixed over the substeps. We obtain approximations $$ w^{[j ]}$$ to $$ v^{[j]} $$ by applying multiple restarted Krylov steps, with orthonormal bases $$ V_m^{[j]}$$ and upper Hessenberg matrices $$ T_m^{[j]}$$. Starting from $$ w^{[0]} = v $$, for $$ j=1,\ldots ,N $$,

$$\begin{aligned} w^{[j]} := S_m^{[j]}(\varDelta t_{j}) w^{[j-1]} = V_m^{[j]}\mathrm{e}^{\sigma \,\varDelta t_{j} T_m^{[j]}} \big (V_m^{[j]}\big )^*w^{[j-1]} = V_m^{[j]}\mathrm{e}^{\sigma \,\varDelta t_{j} T_m^{[j]}}e_1. \end{aligned}$$

The error matrix in the *j*-th step is denoted by

$$\begin{aligned} L_m^{[j]}(\varDelta t_{j}) := E(\varDelta t_{j})-S_m^{[j]}(\varDelta t_{j}), \end{aligned}$$

and the accumulated error by

7.2 $$\begin{aligned} L_m^{\star }(t)v=v^{[N]}-w^{[N]}. \end{aligned}$$

With

$$\begin{aligned} v^{[j]}-w^{[j]}&= E(\varDelta t_j)v^{[j-1]}-S_m^{[j]}(\varDelta t_j) w^{[j-1]} \\&= E(\varDelta t_j)(v^{[j-1]}-w^{[j-1]}) + L_m^{[j]}(\varDelta t_j) w^{[j-1]} \end{aligned}$$

we obtain

$$\begin{aligned} L_m^{\star }(t)v = \sum _{j=1}^N E(\varDelta t_N)\cdots E(\varDelta t_{j+1})\,L_m^{[j]}(\varDelta t_j) w^{[j-1]}. \end{aligned}$$

Recall our premise that $E(\cdot )$ is nonexpansive and assume that the local error is bounded by

7.3 $$\begin{aligned} \Vert L_m^{[j]}(\varDelta t_j)w^{[j]}\Vert _2 \le \mathrm{tol}\cdot \varDelta t_j. \end{aligned}$$

Then,

$$\begin{aligned} \Vert L_m^{\star }(t)v\Vert _2 \le \sum _{j=1}^N \Vert L_m^{[j]}(\varDelta t_j) w^{[j-1]}\Vert _2 \le \mathrm{tol}\sum _{j=1}^N \varDelta t_j = \mathrm{tol}\cdot t. \end{aligned}$$

The term $\Vert L_m^{[j]}(\varDelta t_j)w^{[j]}\Vert _2$ denotes the truncation error of a single substep and is studied in the first part of this paper. We now apply local error estimates to predict acceptable time steps.

*Step size control* For a single substep, the error estimate (${\mathrm {Err}}_{a}$) suggests a step size to satisfy a given error tolerance $\mathrm{tol}$ as

7.4 $$\begin{aligned} \varDelta t_j = \left( \frac{\mathrm{tol}\, \, m!}{\tau ^{[j]}_{m+1,m}\gamma ^{[j]}_m}\right) ^{1/m},\quad j=1,\ldots ,N. \end{aligned}$$

For a local error as in (7.3), we replace $\mathrm{tol}$ by $(\varDelta t_j\,\,\mathrm{tol})$ in (7.4) and obtain

7.5 $$\begin{aligned} \varDelta t_j = \left( \frac{\mathrm{tol}\, \, m!}{\tau ^{[j]}_{m+1,m}\gamma ^{[j]}_m}\right) ^{1/(m-1)},\quad j=1,\ldots ,N. \end{aligned}$$

We remark that $\varDelta t_j$ can be computed together with the construction of the Krylov subspace, therefore, $\tau ^{[j]}_{m+1,m}$ and $\gamma ^{[j]}_m$ are known values at this point. For the corrected Krylov approximation ${S}^+_m(t)v^{[j]}$, see (5.3), the error estimate given in (5.9b) ($p=0$) suggests a local step size of

7.6 $$\begin{aligned} \varDelta t_j = \left( \frac{\mathrm{tol}\, \, (m+1)!}{\big \Vert Av^{[j]}_{m+1}\big \Vert _2\,\tau ^{[j]}_{m+1,m}\gamma ^{[j]}_m}\right) ^{1/m},\quad j=1,\ldots ,N. \end{aligned}$$

The error estimator ${\mathrm {Err}}_{1}$ and estimates given in Sect. 6 cannot be inverted directly to predict the step size. Computing a feasible step size is still possible via heuristic step size control. This approach will be formulated for a general error estimate $\mathrm {Err}$. Ideas of heuristic step size control are given in [20] in general and [47] or [39] for a Krylov approximation of the matrix exponential. For a step with step size $\varDelta t_{j-1}$ and estimated error $\mathrm {Err}^{[j-1]}$, $j=2,\ldots ,N$, a reasonable size for the subsequent step can be chosen as the solution of

7.7 $$\begin{aligned} \varDelta t_{j} = \left( \frac{\varDelta t_{j}\,\,\mathrm{tol}}{\mathrm {Err}^{[j-1]}}\right) ^{1/m} \varDelta t_{j-1} ~~~{\text {resulting in}~~\varDelta t_{j} = \left( \frac{\mathrm{tol}}{\mathrm {Err}^{[j-1]}}\right) ^{1/(m-1)} \varDelta t_{j-1}^{m/(m-1)}.}\nonumber \\ \end{aligned}$$

In (7.7) we only need the evaluation of the error estimate for the previously computed step with step size $\varDelta t_{j-1}$. In the Expokit package [47] the heuristic step size control is used similarly to (7.7) and an initial step size $\varDelta t_1$ is chosen by an a priori choice, which we recall for comparison on numerical examples,

7.8 $$\begin{aligned} \varDelta t_1 = \frac{1}{\Vert H\Vert _{\infty }}\,\left( \frac{\mathrm{tol}\,\, ((m+1)/\mathrm{e})^{m+1}\sqrt{2\,\pi \,(m+1)}}{4\,\Vert H\Vert _{\infty }}\right) ^{1/m}. \end{aligned}$$

In many cases the construction of the Krylov subspace, which is independent of the step size, contributes the largest part to the computational cost. In this case we can improve the choice of $\varDelta t_j$ relatively cheaply in an iterative manner before continuing to time step $j+1$:

7.9 $$\begin{aligned} \begin{aligned} \varDelta t_{j,1}&:= \varDelta t_{j-1}~~\text {or result of}\,(7.5),\\ \varDelta t_{j,l}&:= {\Big ( \frac{\mathrm{tol}}{\mathrm {Err}^{[j,l-1]}}\Big )^{1/(m-1)} \varDelta t_{j,l-1}^{m/(m-1)}},~~~~~ l=2,\ldots ,N_j,\\ \varDelta t_{j}&:=\varDelta t_{j,N_j}.\\ \end{aligned} \end{aligned}$$

By choosing $\mathrm {Err}^{[j,l-1]}$ as an error estimate for the Krylov approximation of the *j*-th step with time step $\varDelta t_{j,l-1}$.

The aim of the iteration (7.9) is to determine a step size $\varDelta t_{j,\infty }$ with $\mathrm {Err}^{[j,\infty ]}=\varDelta t_{j,\infty }\mathrm{tol}$, see (7.3). The convergence behavior of iteration (7.9) depends on the structure of the corresponding error estimate. The idea of the heuristic step size control is based on the asymptotic order of the error for $\varDelta t\rightarrow 0$, which in (7.7) and (7.9) is assumed to be *m*, see (2.11). By substituting the asymptotic order *m* by the effective order $\rho (\varDelta t)+1$, which is introduced in (6.10), the iteration (7.9) could be improved for a step size $\varDelta t$ away from the asymptotic regime. In our practical examples this iteration does not seem to be sensitive with respect to the effective order of the error and converges in a small number of steps using the asymptotic order *m*.

For the following remarks on $T_m$ we neglect the index *j* in $T_m^{[j]}$ to simplify the notation. In the case of a Hermitian matrix $A$ the matrix $T_m$ is symmetric, tridiagonal and real-valued which allows cheap and robust computation of its eigenvalue decomposition. The eigenvalue decomposition of $T_m$ is independent of the step size $\varDelta t$ and allows cheap evaluation of $\mathrm{e}^{\sigma \,\varDelta t\,T_m} e_1$ or $\varphi _1(\sigma \,\varDelta t\,T_m) e_1$ and corresponding error estimates for multiple choices of $\varDelta t$.

For a non-Hermitian matrix $A$ computing $\mathrm {Err}^{[j,l]}$ for multiple choices of *l*, hence different step sizes $\varDelta t_{j,l}$, only leads to slightly larger computational cost, which is usually negligible.

## Numerical considerations and examples

We give an illustration of our theoretical results for two different skew-Hermitian problems in Sect. 8.1, a Hermitian problem in Sect. 8.2, and a non-normal problem in Sect. 8.3. We also compare the performance of different error estimates for practical step size control (Sect. 7) in Sect. 8.1. To show that our error estimate (3.6) is efficient in practice we also compare it with results delivered by the standard package Expokit [47] and a priori error estimates.

### The skew-Hermitian case

For our tests we use different types of matrices.

*Free Schrödinger equation* We consider

8.1 $$\begin{aligned} H=\tfrac{1}{4}\, \text {tridiag}(-1,2,-1) \in {\mathbb {R}}^{n\times n}, \end{aligned}$$

with dimension $n=10\,000$. The matrix *H* is associated with a finite difference or finite element discretization of the one-dimensional negative Laplacian. With $A=H$ and $\sigma =-\mathrm{i}$, in (2.1) we obtain the free Schrödinger equation. The eigenvalue decomposition of *H* is well known, and we can use the discrete sine transform with high precision arithmetic in Matlab to compute the exact solution *E*(*t*)*v*, see (2.1). The starting vector *v* is chosen randomly. To compute the Krylov subspace approximation $S_m(t)v$, see (2.6), we use the eigenvalue decomposition of the tridiagonal matrix $T_m$.

*Discrete Hubbard model* For the description of the Hubbard model we employ a self-contained notation. The Hubbard model first appears in [28] and was further used in many papers and books, e.g., [32, 44]. The Hubbard model is used to describe electron density on a given number of sites, which correspond to Wannier discretization of orbitals, and spin up or down. We consider the following Hubbard Hamiltonian, in second quantization and without chemical potential:

8.2 $$\begin{aligned} H=\frac{1}{2} \sum _{i,j,\sigma } v_{ij} c_{j\sigma }^{\dagger } c_{i\sigma } + \sum _{j,\sigma } U {\hat{n}}_{j\sigma }{\hat{n}}_{j\sigma '}, \end{aligned}$$

where *i*, *j* sum over the number of sites $n_{\text {sites}}$ and the spins $\sigma ,\sigma ' \in \{\uparrow ,\downarrow \}$ where $\sigma '$ is the opposite spin to $\sigma$. The entries $v_{ij}$ with $i,j=1,\ldots ,n_{\text {sites}}$ describe electron hopping from site *i* to *j*. In (8.2), the notation $c_{j\sigma }^\dagger c_{i\sigma }$ describes the 2nd quantization operator and ${\hat{n}}_{j\sigma }=c_{j\sigma }^\dagger c_{j\sigma }$ the occupation number operator. For details on the notation in (8.2) we can recommend several references, e.g., [28, 29, 32, 44].

For our tests we model 8 electrons at 8 sites ($n_{\text {sites}}=8$) with spin up and down for each site, this leads to 16 possible states for electrons. Such an electron distribution is also referred to as half-filled in the literature. We also restrict our model by considering the number of electrons with spin up and down to be fixed as $n_{\text {sites}}/2$. This leads to $n=(\text {binomial}(8,4))^2=4900$ considered occupation states which create a discrete basis. For the numerical implementation of the basis we consider 16-bit integers for which each bit describes a position which is occupied in case the bit is equal to 1 or empty otherwise. The set of occupation states can be ordered by the value of the integers which leads to a unique representation of the Hubbard Hamiltonian (8.2) by a matrix $H\in {\mathbb {C}}^{n\times n}$. Such an implementation of the Hubbard Hamiltonian is also described in [29, Section 3].

In our test setting we use $U=5$ and parameter-dependent values for electron hopping $v_{ij}=v_{ij}(\omega )\in {\mathbb {C}}$ with $\omega \in (0,2\pi ]$:

$$\begin{aligned}&v_{11}=v_{88}=-1.75,\quad v_{jj}=-2\quad \text {for}\quad j=2,\ldots ,7,\\&v_{j,j+1}={\bar{v}}_{j+1,j}=-\cos \omega +\mathrm{i}\,\sin \omega \quad \text {for}\quad j=1,\ldots ,7\quad \text {and}\quad v_{ij}=0\quad \text {otherwise}. \end{aligned}$$

For this choice of $v_{ij}(\omega )$ we obtain a Hermitian matrix $H_\omega \in {\mathbb {C}}^{n\times n}$ with 43,980 nonzero entries (for a general choice of $\omega$) and $\text {spec}(H_\omega )\subseteq (-19.1,8.3)$. The spectrum of $H_\omega$ is independent of $\omega$.

A relevant application where the Hubbard Hamiltonian (8.2) is of importance is the simulation of oxide solar cells with the goal of finding candidates for new materials promising a gain in the efficiency of the solar cell, see [21]. The study of solar cells considers time-dependent electron hoppings $v_{ij}=v_{ij}(t)$ to model time-dependent potentials which lead to Hamiltonian matrices *H*(*t*). The time-dependent Hamiltonian can be parameterized via $\omega$. Time propagation of a linear, non-autonomous ODE system can be approximated by Magnus-type integrators which are based on one or more evaluations of matrix exponentials applied to different starting vectors at several times *t*, see for instance [5, 6]. Our test setting for the Hubbard Hamiltonian with arbitrary $\omega$ is then obtained by (2.1) with the matrix $A=H_\omega$ as described above and $\sigma =-\mathrm{i}$.

In the following Sect. 8.1 we focus on the skew-Hermitian case. For tests on the Hermitian case see Sect. 8.2 below.

**Fig. 1:**
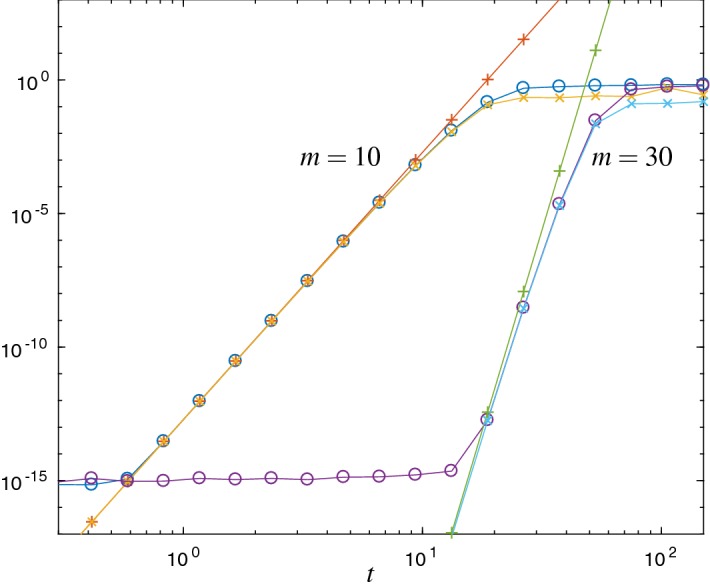
Error $\Vert L_m(t)v\Vert _2$ ($\circ$) and the error estimates ${\mathrm {Err}}_{1}$ ($\times$) and ${\mathrm {Err}}_{a}$ ($+$) for the free Schrödinger problem and Krylov subspace dimensions $m=10$ and $m=30$. ${\mathrm {Err}}_{a}$ is an upper bound for the error, and both estimates show the correct asymptotical behavior. Due to round-off error, for $m=30$ the observed effective order is less clear than for $m=10$

**Fig. 2:**
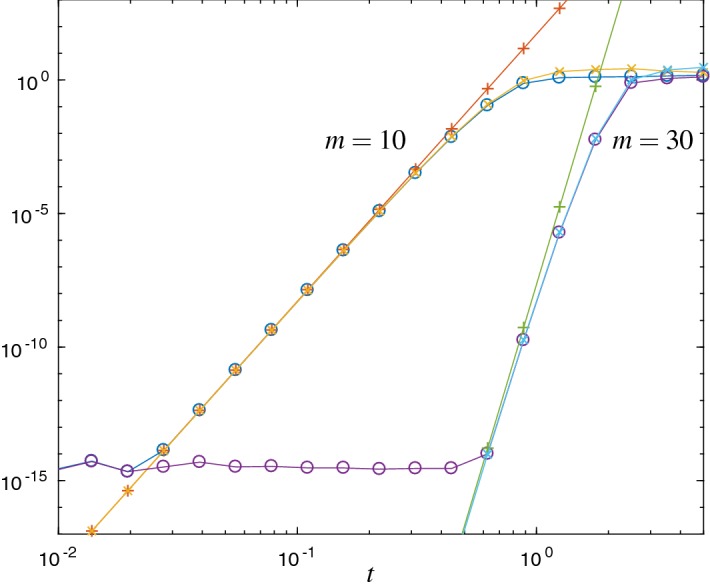
Error $\Vert L_m(t)v\Vert _2$ ($\circ$) and the error estimates ${\mathrm {Err}}_{1}$ ($\times$) and ${\mathrm {Err}}_{a}$ ($+$) for the Hubbard Hamiltonian with $\omega =0.123$ and Krylov subspace dimensions $m=10$ and $m=30$. This shows the same behavior as in Fig. 1

*Verification of upper error bound* In the following Figs. 1 and 2 we compare the error $\Vert L_m(t)v\Vert _2$ with the error estimates ${\mathrm {Err}}_{1}$ and ${\mathrm {Err}}_{a}$. Figure 1 refers to the matrix (8.1) of the free Schrödinger problem and Fig. 2 to the Hubbard Hamiltonian (8.2) with $\omega =0.123$. For both cases we show results with Krylov subspace dimensions $m=10$ and $m=30$, respectively.

We observe that the error estimate ${\mathrm {Err}}_{1}$ is a good approximation to the error, but it is not an upper bound in general. In contrast, ${\mathrm {Err}}_{a}$ is a proven upper error bound. Up to round-off error, for $m=10$ we observe the correct asymptotic behavior of ${\mathrm {Err}}_{a}$ and ${\mathrm {Err}}_{1}$. For larger choices of *m* the asymptotic regime starts at time steps for which the error is already close to round-off precision. Therefore, for larger choices of *m*, the Krylov approximation, as a time integrator, cannot achieve its full order for typical time steps in double precision.

The matrix (8.1) has been scaled such that $\text {spec}(H)\subseteq (0,1)$ and $\Vert H\Vert _2\approx 1$. In accordance with (7.1) stagnation of the error is observed for times $t \lessapprox m$, see Fig. 1.

**Fig. 3:**
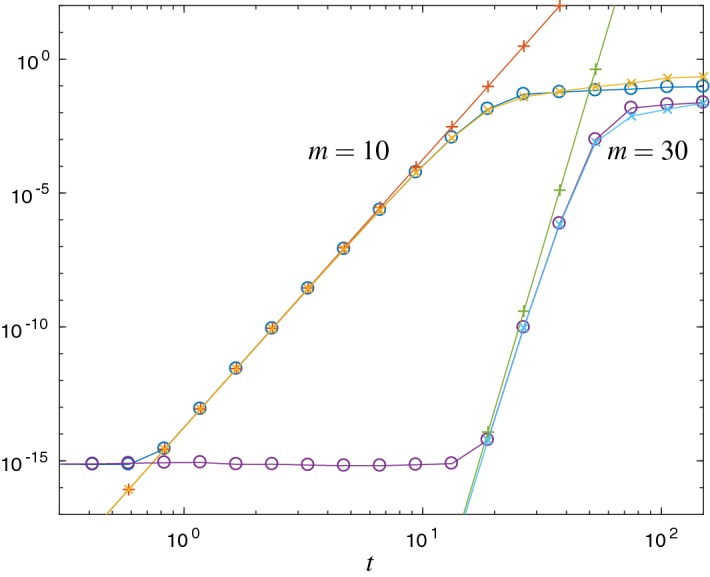
Error $\Vert L_m^1(t)v\Vert _2$ ($\circ$) and the error estimates ${\mathrm {Err}}_{1}$ ($\times$) and ${\mathrm {Err}}_{a}$ ($+$) for the free Schrödinger problem and Krylov subspace dimension $m=10$ and $m=30$

**Fig. 4:**
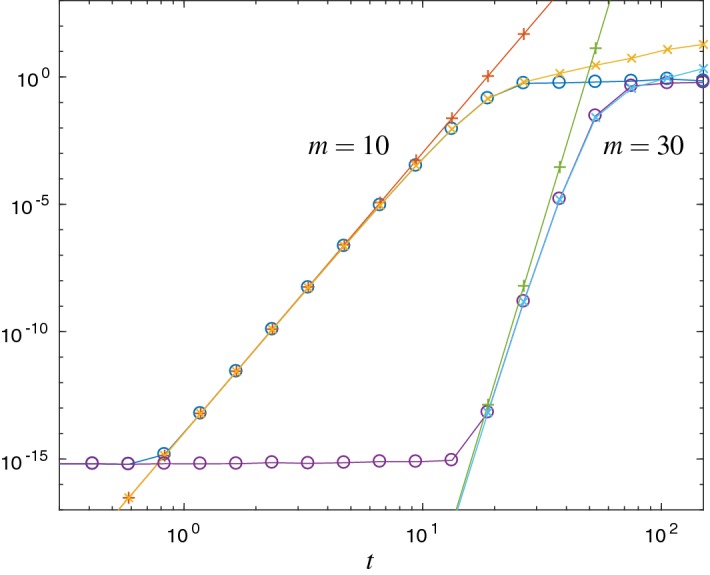
Error $\Vert L^{+}_m(t) v\Vert _2$ ($\circ$) and the error estimates ${\mathrm {Err}}_{1}$ ($\times$) and ${\mathrm {Err}}_{a}$ ($+$) for the free Schrödinger problem and Krylov subspace dimension $m=10$ and $m=30$

We verify the error estimates in the skew-Hermitian setting of the free Schrödinger Eq. (8.1) for the standard Krylov approximation of the $\varphi _1$ function in Fig. 3 and the corrected Krylov approximation of the matrix exponential function in Fig. 4. In Fig. 3 the error estimator ${\mathrm {Err}}_{1}$ refers to formula (5.8a) and ${\mathrm {Err}}_{a}$ shows the upper error bound (4.4b) from Theorem 2, both for the case $p=1$. In Fig. 4, ${\mathrm {Err}}_{1}$ is from formula (5.8b) and ${\mathrm {Err}}_{a}$ denotes the upper error bound (5.9b) from Theorem 3, both for the case $p=0$.

**Fig. 5:**
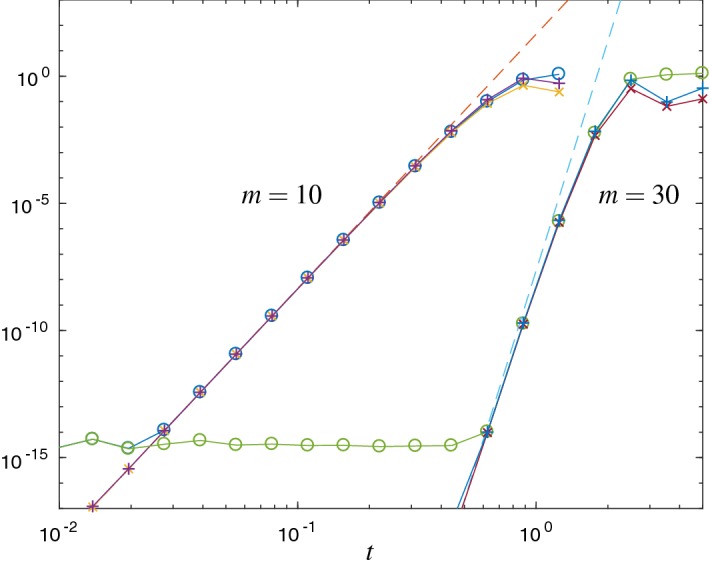
Error $\Vert L_m(t) v\Vert _2$ ($\circ$) and the error estimates based on the Hermite quadrature ($\times$) and improved Hermite quadrature ($+$), see (6.3) and (6.5), for the Hubbard Hamiltonian with $m=10$ and $m=30$. The dashed lines show the error estimate ${\mathrm {Err}}_{a}$

*Illustration of defect-based quadrature error estimates from Section* 6 We first illustrate the performance of the estimates based on Hermite quadrature according to (6.3) and improved Hermite quadrature according to (6.5) for the Hubbard model, see Fig. 5. Both estimates are asymptotically correct, whereas the improved quadrature (6.5) is slightly better for larger time steps *t*, with the drawback of one additional matrix–vector multiplication. (See Remark 7 below for cost efficiency of more expensive error estimates.)

**Fig. 6:**
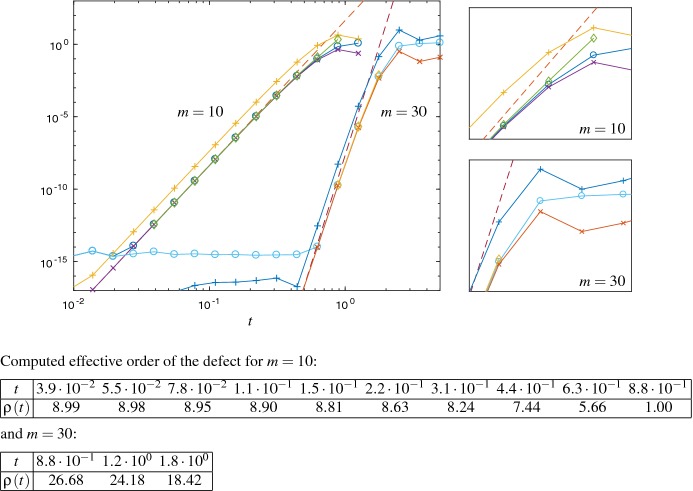
The upper left plot shows the error $\Vert L_m(t) v\Vert _2$ ($\circ$), the generalized residual estimate (6.6) ($+$) and the error estimates based on the Hermite quadrature (6.3) ($\times$), and the effective order quadrature (6.9) ($\Diamond$) for the Hubbard Hamiltonian with $m=10$ and $m=30$. The dashed lines show the error estimate ${\mathrm {Err}}_{a}$. On the right-hand side the graphics show a detail from the error plots to illustrate the inequalities (6.8). The table on the bottom shows the computed effective order of the defect for $m=10$ and $m=30$ which is used for the effective order quadrature

Figure 6 refers to the generalized residual estimate (6.6), and estimates based on the effective order quadrature according to Remark 5, and the Hermite quadrature (6.3). For our test problems the assumptions from Sect. 6 on the defect and its effective order are satisfied for a significant range of values of *t*. We also observe that the inequalities (6.8) are satisfied. The effective order and Hermite quadrature estimates behave in an asymptotically correct way, while the generalized residual estimate leads to an upper error bound which is, however, not sharp for $t\rightarrow 0$.

For the skew-Hermitian case use $\sigma =-\mathrm{i}$ and $T_m\in {\mathbb {R}}^{m\times m}$ in (6.10) to obtain

$$\begin{aligned} \rho (t)=t\,(T_m)_{m-1,m}\,\text {Re}\bigg ( \frac{-\mathrm{i}\,(\mathrm{e}^{-\mathrm{i}\,t\,T_m}e_1)_{m-1}}{(\mathrm{e}^{-\mathrm{i}\,t\,T_m}e_1)_m} \bigg ). \end{aligned}$$

For computing the effective order we only consider time steps $\rho (t)>0$, and where $\rho$ appears indeed to be monotonically decreasing over the computed discrete time steps. This restriction is compatible with our assumptions in Sect. 6.

*Corrected Krylov approximation and mass conservation* We remark that error estimates for the corrected Krylov approximation usually require one additional matrix–vector multiplication, and applying a standard Krylov approximation of dimension $m+1$ seems to be a more favorable choice in our approach to error estimation.

The Krylov approximation of the matrix exponential conserves the mass for the skew-Hermitian case in contrast to the corrected Krylov approximation. Whether this is a real drawback of the corrected Krylov approximation depends on the emphasis placed on mass conservation. In the following examples we focus on the standard Krylov approximation, with some exceptions which serve for comparisons with the original Expokit code, which is based on the corrected Krylov approximation.

In exact arithmetic we obtain mass conservation for the skew-Hermitian case: For the case $\Vert v\Vert _2=1$ and the standard Krylov approximation $S_m(t)v$ we have

8.3 $$\begin{aligned} \Vert S_m(t)v\Vert _2 = \Vert V_m \mathrm{e}^{-\mathrm{i}\,t\,T_m}e_1\Vert _2 = e_1^*\mathrm{e}^{\mathrm{i}\,t\,T_m} V_m^*V_m\mathrm{e}^{-\mathrm{i}\,t\,T_m}e_1=1. \end{aligned}$$

The requirement $V_m^*V_m=I$ is essential to obtain mass conservation in (8.3). In computer arithmetic the loss of orthogonality of the Krylov basis $V_m$ has been studied earlier, see also [40]. To preserve the property of mass conservation a reorthogonalization, see [43], may be advisable in this case.

*Krylov approximation of the matrix exponential in computer arithmetic* It has been shown in [11, 13, 19] that a priori error estimates for the Krylov approximation of the matrix exponential remain valid also taking account of affects of arithmetic. Such results imply that in general in computer arithmetic the convergence of the Krylov approximation is not precluded and round-off errors are not critical. In practice round-off errors may in some cases lead to a delay of convergence which can make a reorthogonalization relevant. Stability of the Krylov approximation has been discussed by many authors, see also [38], but is not further discussed here in detail. In the next paragraph we will give an argument, following [13], that the a posteriori error estimates which are the topic of this work are robust with respect to round-off errors.

We recall that the Krylov subspace constructed in computer arithmetic satisfies the Krylov identity (2.2) with a small perturbation, see also [40] for the Lanczos case and [8, 51] for the Arnoldi case, which can both be extended to complex-valued problems using results from [22]. Following results from [13] we conclude that a small perturbation of the Krylov identity leads to a small perturbation of the defect (residual) $\delta _m(t)$ in (3.1a) and the integral representation of the error in (3.1b). Thus the error estimates given in Sect. 6 remain stable with respect to round-off.

We further use that by construction the computed $T_m$ is still upper Hessenberg with a positive lower diagonal and in the Lanczos case also real-valued and symmetric. Then following Proposition 6 in the Hermitian (Lanczos) case, the integral representation of the error in (3.1b) results in the upper error bound ${\mathrm {Err}}_{1}$, which is not critically affected by round-off errors. For the upper bound ${\mathrm {Err}}_{a}$ we further assume that spectral properties of (3.4) still hold mutatis mutandis under a small perturbation, see [41] for such results for the Lanczos case, to obtain stability of this upper error bound also with round-off.

*Numerical tests for step size control* The idea of choosing discrete time steps for the Krylov approximation is described in Sect. 7. The following tests are applied to the matrix exponential of the Hubbard Hamiltonian. We first clarify the notation used for our test setting.

- *Expokit and*$\hbox {Expokit}^{\star }$ The original Expokit code uses the corrected Krylov approximation with heuristic step size control and an error estimator which is based on the error expansion (5.1), see [47, Algorithm 3.2] for details. Since the standard Krylov approximation is not part of the Expokit package, we have slightly adapted the code and its error estimate such that the standard Krylov approximation is used. We refer to the adapted package as $\hbox {Expokit}^{\star }$. With $\hbox {Expokit}^{\star }$ our comparison can be drawn with the standard Krylov approximation which may in some cases be the method of choice as discussed above.
- *Step size based on*${\mathrm {Err}}_{a}$ In another test code the upper error bound ${\mathrm {Err}}_{a}$ from Theorem 1 is used. With ${\mathrm {Err}}_{a}$ we obtain proven upper bounds on the error and reliable step sizes (7.5).
- By *gen.res*, *eff.o.quad*, and $$ Err _1$$ we refer to the generalized residual estimate (6.6), the effective order quadrature (6.9), and ($${\mathrm {Err}}_{1}$$), respectively. Because these error estimates cannot be inverted directly we need to apply heuristic ideas for the step size control, see (7.7). In addition, we use the iteration (7.9) to improve step sizes. For the test problems we have solved, iteration (7.9) converges in less than 2 iterations for $$m=10$$ or less than 5 iterations for $$m=30$$. We simply choose $$N_j=5$$ for our tests.
- The *a priori estimates* (7.8), [24, Theorem 4] *and* [34, eq. (20)] are given in the corresponding references. Formula (7.8) taken from the Expokit code directly provides a step size. In [34, eq. (20)] the computation of the step size is described. For the error estimate given in [24, Theorem 4] we apply Newton iteration to determine an appropriate step size. For tests on the Hubbard model we use $(\lambda _{\text {max}} - \lambda _{\text {min}}) = 27.4$ as suggested in the description of the Hubbard Hamiltonian.

In Remark 7 below we also investigate the following variants:

- *Step size based on*${\mathrm {Err}}_{a}^+$ By ${\mathrm {Err}}_{a}^+$ we denote the upper error bound for the corrected Krylov approximation as given in Theorem 3 with $p=0$. The corresponding step size is given by (7.6).
- By *i.H.quad* we refer to the improved Hermite quadrature (6.5). Similarly to other quadrature error estimates we use heuristic step size control and iteration (7.9) to determine adequate step sizes.

#### Remark 6

In the Expokit code the step sizes are rounded to 2 digits in every step. Rounding the step size can give too large errors in some steps. This makes it necessary to include safety parameters in Expokit which on the other hand slow down the performance of the code. It seems advisable to avoid any kind of rounding of step sizes.

In Table 1 we compare the total time step *t* for the Krylov approximation with $m=10$ and $m=30$ after $N=10$ steps obtained with the different step size control strategies. For the local error we choose the tolerance $\mathrm{tol}=10^{-8}$. The original Expokit code seems to give larger step sizes, but it also uses a larger number of matrix–vector multiplications, see Remark 7. The error estimate ${\mathrm {Err}}_{a}$ leads to optimal step sizes for $m=10$ and close to optimal step sizes for $m=30$. For any choice of *m* the error estimate ${\mathrm {Err}}_{a}$ gives reliable step sizes. The generalized residual estimate overestimates the error and, therefore, step sizes are smaller. The effective order quadrature and ${\mathrm {Err}}_{1}$ give optimal step sizes. With the assumptions from Sect. 6 (which apply to our test examples), the generalized residual estimate and effective order quadrature give reliable step sizes. For the error estimate ${\mathrm {Err}}_{1}$ we do not have results on the reliability of the step sizes since the error estimate ${\mathrm {Err}}_{1}$ does not lead to an upper bound of the error in general. The tested a priori estimates (7.8), [24, Th. 4], and [34, (20)] overestimate the error and lead to precautious step size choices. For all the tested versions the accumulated error $L_m^{\star }$ (see (7.2)) satisfies $\Vert L_m^{\star } v\Vert _2/t \le \mathrm{tol}$.

**Table 1:** The displayed step size *t* is the sum of $N=10$ substeps computed by different versions of step size control, as described above

	Expokit	$\text {Expokit}^{\star }$	${\mathrm {Err}}_{a}$	gen.res	eff.o.quad	${\mathrm {Err}}_{1}$	(7.8)	[24, Th. 4]	[34, (20)]
$$m=10$$									
*t*	0.9020	0.6850	0.8468	0.6568	0.8488	0.8489	0.1918	0.4918	0.6879
*N*	10	10	10	10	10	10	10	10	10
# m–v	110	100	100	100	100	100	100	100	100
$\Vert L_m^{\star } v\Vert _2/t$	$3.5\times 10^{-09}$	$2.9\times 10^{-09}$	$9.8\times 10^{-09}$	${1.0\times 10^{-09}}$	$1.0\times 10^{-08}$	$1.0\times 10^{-08}$	$3.0\times 10^{-14}$	$7.5\times 10^{-11}$	$1.5\times 10^{-09}$
$$m=30$$									
*t*	8.5700	8.2500	9.7248	9.0091	10.2127	10.2222	2.1131	8.2642	8.8111
*N*	10	10	10	10	10	10	10	10	10
# m–v	310	300	300	300	300	300	300	300	300
$\Vert L_m^{\star } v\Vert _2/t$	$2.6\times 10^{-10}$	$2.9\times 10^{-10}$	$2.6\times 10^{-09}$	${3.5\times 10^{-10}}$	$9.5\times 10^{-09}$	$9.7\times 10^{-09}$	$2.9\times 10^{-15}$	$3.4\times 10^{-11}$	$1.9\times 10^{-10}$

In the top table we show the results for $m=10$, in the bottom table for $m=30$, both for tolerance $\mathrm{tol}=10^{-8}$, for the Hubbard Hamiltonian

**Table 2:** With a test setting similar to Table 1, we now compute up to a fixed time $t=0.3$ and choose the number *N* of steps according to the step size control

${m=10}$	Expokit	$\text {Expokit}^{\star }$	${\mathrm {Err}}_{a}$	gen.res	${\mathrm {Err}}_{1}$	(7.8)	[24, Th. 4]	[34, (20)]
*t*	0.3	0.3	0.3	0.3	0.3	0.3	0.3	0.3
*N*	2	2	1	1	1	2	1	1
# m–v	62	60	17	30	30	60	30	30
$\Vert L_m^{\star } v\Vert _2/t$	$8.4\times 10^{-15}$	$8.4\times 10^{-15}$	$1.0\times 10^{-09}$	$9.7\times 10^{-15}$	$9.7\times 10^{-15}$	$1.0\times 10^{-14}$	$9.7\times 10^{-15}$	$9.7\times 10^{-15}$

We use a tolerance $\mathrm{tol}=10^{-8}$ and $m=30$. For this problem we see a significant reduction in the number of matrix–vector multiplications used for the estimate ${\mathrm {Err}}_{a}$ by the stopping critera described in the text

Apart from step size control, the upper error bound ${\mathrm {Err}}_{a}$ can be used on the fly to test if the dimension of the Krylov subspace is already sufficiently large to solve the problem in a single time step with the required accuracy. For our test problems this stopping criterion is applied to the ${\mathrm {Err}}_{a}$ estimate. We refer to Table 2, in which we observe the Krylov method with error estimate ${\mathrm {Err}}_{a}$ to stop after 17 steps instead of computing the full Krylov subspace of dimension 30. In comparison, the original Expokit package needs a total of 62 matrix–vector multiplications.

#### Remark 7

Error estimates for the corrected Krylov approximation or improved error estimates such as the improved Hermite quadrature (6.5) require additional matrix–vector multiplications. Instead of investing computational effort in improving the error estimate, one may as well increase the dimension of the standard Krylov subspace. For comparison we test the original Expokit code, the corrected Krylov approximation with error estimate ${\mathrm {Err}}_{a}^+$ and the improved Hermite quadrature (6.5) with Krylov subspace $m-1$. Table 3 shows that a standard Krylov approximation with dimension *m* leads to better results, although all considered versions use the same number of matrix–vector multiplications. Since the reliability of error estimates such as ${\mathrm {Err}}_{a}$ has been demonstrated earlier, it appears that additional cost to improve the error estimate is not justified.

**Table 3:** All variants shown use exactly *m* matrix–vector multiplications

	Expokit	${\mathrm {Err}}_{a}^+$	i.H.quad	${\mathrm {Err}}_{a}$	eff.o.quad	${\mathrm {Err}}_{1}$
$$m=10$$						
*t*	0.6620	0.7828	0.5863	0.8346	0.8366	0.8368
*N*	10	10	10	10	10	10
# m–v	100	100	100	100	100	100
$\Vert L_m^{\star } v\Vert _2/t$	$4.1\times 10^{-09}$	$8.8\times 10^{-09}$	$1.0\times 10^{-08}$	$9.8\times 10^{-09}$	$1.0\times 10^{-08}$	$1.0\times 10^{-08}$
$$m=30$$						
*t*	8.1900	9.5763	9.6591	9.7482	10.2378	10.2473
*N*	10	10	10	10	10	10
# m–v	100	100	100	100	100	100
$\Vert L_m^{\star } v\Vert _2/t$	$3.6\times 10^{-10}$	$2.7\times 10^{-09}$	$9.2\times 10^{-09}$	$2.6\times 10^{-09}$	$9.5\times 10^{-09}$	$9.7\times 10^{-09}$

Whereas Expokit, improved Hermite quadrature (i.H.quad) and ${\mathrm {Err}}_{a}^+$ imply higher cost for the error estimate, the other codes ${\mathrm {Err}}_{a}$, effective order quadrature (eff.o.quad) and ${\mathrm {Err}}_{1}$ use standard Krylov subspaces and do not spend additional matrix–vector multiplications on error estimates

### The Hermitian case

To obtain a more complete picture, we also briefly consider the case of a Hermitian matrix $A=H$ with $\sigma =1$ in (2.1). Such a model is typical of the discretization of a parabolic PDE. Thus, the result may depend on the regularity of the initial data, which is chosen to be random in our experiments.

*Heat equation* To obtain the heat equation in (2.1) we choose $A=H$ in (8.1) and $\sigma =-1$. Details on the test setting are already given in Sect. 8.1.

**Fig. 7:**
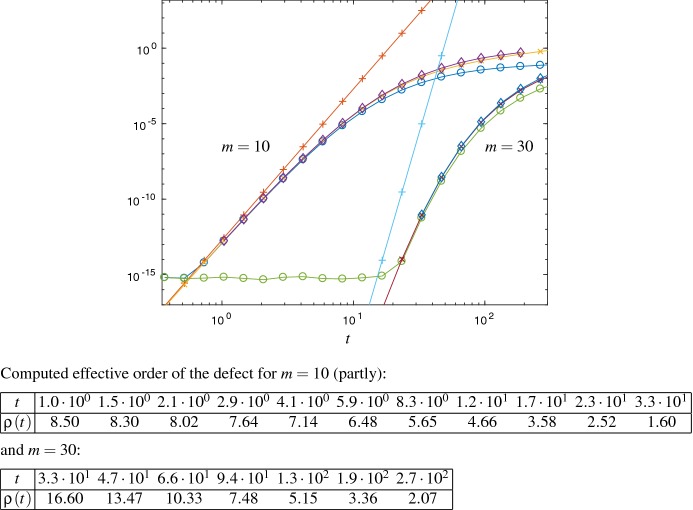
Error $\Vert L_m(t)v\Vert _2$ ($\circ$), the error estimates ${\mathrm {Err}}_{1}$ ($\times$) and ${\mathrm {Err}}_{a}$ ($+$) and the error estimate based on the effective order quadrature (6.9) ($\Diamond$) for the heat equation with $m=10$ and $m=30$. The tabular on the bottom shows some of the computed values for the effective order

For the heat equation, *H* given in (8.1), we can also verify the error estimates, see Fig. 7. In comparison to the skew-Hermitian case we do not observe a large time regime for which the error is of the asymptotic order *m*. As shown in Proposition 6 we do obtain an upper error bound using ${\mathrm {Err}}_{1}$ for the heat equation.

Similarly to the skew-Hermitian case, we can also apply the effective order quadrature according to Remark 5 to the Hermitian case. Use $\sigma =-1$ and $T_m\in {\mathbb {R}}^{m\times m}$ in (6.10) to obtain

$$\begin{aligned} \rho (t)=-t\,\bigg ((T_m)_{m,m} + (T_m)_{m,m-1}\, \frac{(\mathrm{e}^{-t\,T_m}e_1)_{m-1}}{(\mathrm{e}^{-t\,T_m}e_1)_m} \bigg ). \end{aligned}$$

For computing the effective order we only consider time steps $\rho (t)>0$, and where $\rho$ appears indeed to be monotonically decreasing over the computed discrete time steps. This restriction is compatible with our assumptions in Sect. 6.

### A non-normal problem

For a more general case we consider a convection–diffusion equation (see [14, 36]).

8.4 $$\begin{aligned}&\partial _t u = \varDelta u - \tau _1 \partial _{x_1} u - \tau _2 \partial _{x_2} u,\nonumber \\&\quad \tau _1,\tau _2\in {\mathbb {R}},\quad u=u(t,x),~~t\ge 0,\quad x\in \varOmega =[0,1]^3,\nonumber \\&u(0,x)=v(x)\quad \text {for}\quad x\in \varOmega ,\quad u(t,x)=0\quad \text {for}\quad x\in \partial \varOmega . \end{aligned}$$

Following [14, 36] we use a central finite difference scheme to discretize the partial differential operator in (8.4). The grid is chosen uniformly with $(n+2)^3$ points and mesh width $h=1/(n+1)$. The dimension *N* of the discrete operator is $N=n^3$. Choosing $n=15$ we obtain $N=3357$. The discretized operator is given by

8.5 $$\begin{aligned} A&=( I_{n\times n} \otimes (I_{n\times n} \otimes C_1) ) + ( B \otimes I_{n\times n} + I_{n\times n}\otimes C_2 ) \otimes I_{n\times n} )\in {\mathbb {R}}^{N\times N},\quad \text {with} \nonumber \\ B&=\tfrac{1}{h^2} \text {tridiag}(1,-2,1)\in {\mathbb {R}}^n,\nonumber \\&\quad C_i=\tfrac{1}{h^2} \text {tridiag}(1+\mu _i,-2,1-\mu _i)\in {\mathbb {R}}^n,\quad i=1,2, \end{aligned}$$

and $\mu _i=\tau _i\,(h/2)$. The spectrum of the non-normal matrix *A* in (8.5) (see [36]) satisfies

$$\begin{aligned} \text {spec}(A)&\subseteq \tfrac{1}{h^2}\,[-6-2\cos (\pi \,h)\text {Re}(\theta ),-6+2\cos (\pi \,h)\text {Re}(\theta )]\\&\times \tfrac{1}{h^2}\,\mathrm{i}\,[-2\cos (\pi \,h)\text {Im}(\theta ),2\cos (\pi \,h)\text {Im}(\theta )]. \end{aligned}$$

with $\theta = 1 + \sqrt{1-\mu _1^2} + \sqrt{1-\mu _2^2}$. Therefore, the eigenvalues are complex-valued if at least one $\mu _i>1$. The matrix *A* depends on the parameters $\mu _i$, correspondingly $\tau _i$, for which we consider two different cases,

8.6 $$\begin{aligned} \mu _1=0.9, \mu _2=1.1,\quad \text {with}\quad \text {spec}(h^2\,A) \subseteq [-9,-3] \times \mathrm{i}[-1,1], \end{aligned}$$

and

8.7 $$\begin{aligned} \mu _1=\mu _2=10,\quad \text {with}\quad \text {spec}(h^2\,A) \subseteq [-8,-4] \times \mathrm{i}[ -39,39]. \end{aligned}$$

In the following numerical experiments we apply the Krylov approximation to $\mathrm{e}^{t\,A}v$ ($\sigma =1$ in (2.1)) for different time steps *t* and starting vector $v=(1,\ldots ,1)^*\in {\mathbb {R}}^{N}$ as in [36]. For non-normal *A* we use the Arnoldi method based on a modified Gram–Schmidt procedure (see [46, Algorithm 6.2]) to generate the Krylov subspace.

**Fig. 8:**
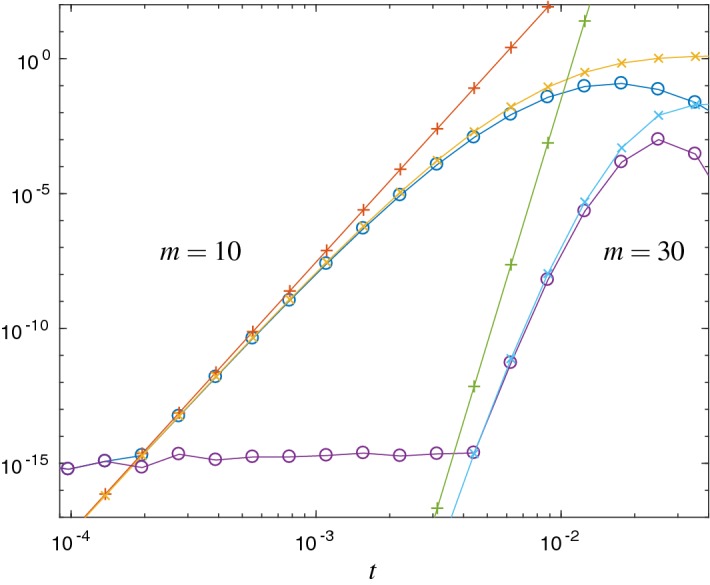
Error $\Vert L_m(t)v\Vert _2$ ($\circ$) and the error estimates ${\mathrm {Err}}_{1}$ ($\times$) and ${\mathrm {Err}}_{a}$ ($+$) for the convection–diffusion problem (8.5) with $\mu _1=0.9$ and $\mu _2=1.1$ and Krylov subspace dimensions $m=10$ and $m=30$

**Fig. 9:**
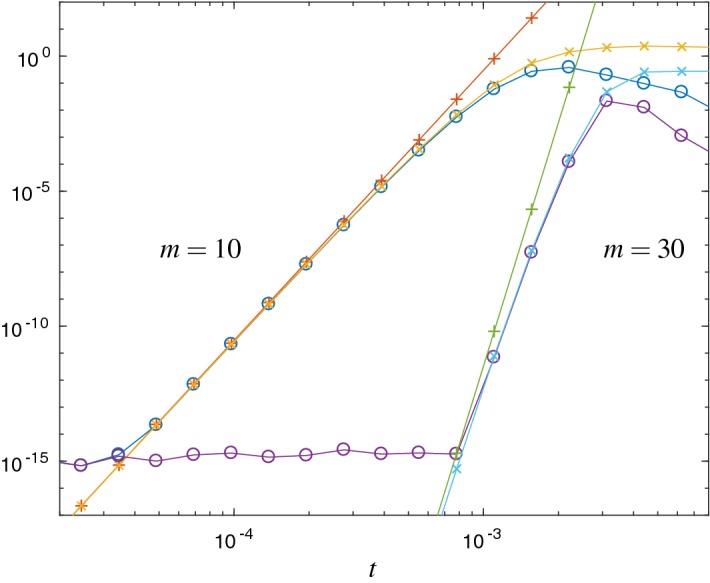
Error $\Vert L_m(t)v\Vert _2$ ($\circ$) and the error estimates ${\mathrm {Err}}_{1}$ ($\times$) and ${\mathrm {Err}}_{a}$ ($+$) for the convection–diffusion problem (8.5) with $\mu _1=\mu _2=10$ and Krylov subspace dimensions $m=10$ and $m=30$

The error estimates ${\mathrm {Err}}_{a}$ and ${\mathrm {Err}}_{1}$ are compared to the exact error norm $\Vert L_m(t) v\Vert _2$ in Fig. 8 for the case (8.6) and in Fig. 9 for the case (8.7). As shown in Theorem 1 the error estimate ${\mathrm {Err}}_{a}$ constitutes an upper error bound. The error estimate ${\mathrm {Err}}_{1}$ gives a good approximation of the error but has not been proven to give an upper bound in general.

Compared to (8.7), the spectrum for (8.6) is closer to the Hermitian case. The spectrum for (8.7), on the other hand, is dominated by large imaginary parts similarly as in the skew-Hermitian case.

In Fig. 8 we observe effects similar to the Hermitian case. The asymptotic order *m* of the error does not hold for a large time regime, and the error estimate ${\mathrm {Err}}_{a}$ is not as sharp as in the skew-Hermitian case. On the other hand, in Fig. 9, we observe that the performance of the error estimates is closer to the skew-Hermitian case. Therefore, the upper error bound ${\mathrm {Err}}_{a}$ is sharp for a larger range of time steps. As already observed for the Hermitian and skew-Hermitian cases, the error of the Krylov approximation is closer to its asymptotic order *m* for smaller choices of *m*.

## Summary and outlook

We have studied a new reliable error estimate ${\mathrm {Err}}_{a}$ for Krylov approximations to the matrix exponential and $\varphi$-functions. This error estimate constitutes an upper bound on the error, and it can be computed on the fly at nearly no additional cost. The Krylov process can be stopped as soon as the error estimate satisfies a given tolerance. ${\mathrm {Err}}_{a}$ is asymptotically correct for $t \rightarrow 0$ and very tight in the asymptotic regime. Our numerical experiments illustrate that the asymptotic regime is more relevant for the skew-Hermitian case (compared to the Hermitian case) and for a smaller choice of *m* and tolerances. The non-normal examples seem to be in between the skew-Hermitian and Hermitian cases.

In our numerical experiments the defect (residual) is seen to behave nicely close to the asymptotic regime and the generalized residual estimate is observed to constitute an upper bound. The generalized residual estimate can be tightened by applying an effective order quadrature.

For the Hermitian case we have shown that the error estimate ${\mathrm {Err}}_{1}$ constitutes an upper bound and, compared to other error estimates, seems to be the most appropriate choice for Hermitian problems.

Step size control for a simple restarted scheme is an important application. The upper error bound ${\mathrm {Err}}_{a}$ is an appropriate tool for this task, since the optimal step size for a given tolerance can be computed directly. This is not the case for other error estimates for the Krylov approximation, which usually employ heuristic schemes to compute optimal step sizes in the restarting approach. We have shown that the step size can be cheaply improved by using a heuristic step size approach in an iterative manner. Also the use of a priori bounds is not optimal in most cases.

## Footnotes

1 In this case the matrix *A* is usually named *H* (Hamiltonian).

2 Here, ${e_m = (0,\ldots ,0,1)^*\in {\mathbb {C}}^m}$, and in the sequel we also denote ${e_1 = (1,0,\ldots ,0)^*\in {\mathbb {C}}^m}$.

3 See also Sect. 6 below.

4 In the sequel, the argument of $\delta _m(\cdot )$ is again denoted by *t* instead of *s*.

5 In the setting of [3] (higher-order splitting methods) such an improved error estimate was not taken into account since it cannot be evaluated with reasonable effort in that context.
